# CCN2/CTGF tip the balance of growth factors towards TGF-β2 in primary open-angle glaucoma

**DOI:** 10.3389/fmolb.2023.1045411

**Published:** 2023-05-11

**Authors:** Andrea E. Dillinger, Sabrina Kuespert, Amin A. Seleem, Jakob Neuendorf, Magdalena Schneider, Rudolf Fuchshofer

**Affiliations:** ^1^ Institute of Human Anatomy and Embryology, University of Regensburg, Regensburg, Germany; ^2^ Zoology Department, Faculty of Science, Sohag University, Sohag, Egypt; ^3^ Biology Department, Faculty of Science and Arts, Al Ula, Taibah University, Almadinah Almunawwarah, Saudi Arabia

**Keywords:** primary open angle glaucoma, ciliary body development, growth factors, trabecular meshwork, fibrosis, extracellular matrix, bone morphogenetic proteins

## Abstract

TGF-β2 is the predominant TGF-β isoform within the eye. One function of TGF-β2 is to provide the eye with immune protection against intraocular inflammation. The beneficial function of TGF-β2 within the eye must be under tight control of a network of different factors. A disbalance of the network can result in different eye diseases. In Primary Open-Angle Glaucoma (POAG), one of the leading causes of irreversible blindness worldwide, TGF-β2 is significantly elevated in the aqueous humor and antagonistic molecules like BMPs are reduced. The changes provoke an altering of the quantity and quality of the extracellular matrix and the actin cytoskeleton in the outflow tissues, leading to an increased outflow resistance and thereby to an increased intraocular pressure (IOP), the major risk factor for primary open-angle glaucoma. The pathologic effect of TGF-β2 in primary open-angle glaucoma is mainly meditated by CCN2/CTGF. CCN2/CTGF can modulate TGF-β and BMP signaling by direct binding. The eye specific overexpression of CCN2/CTGF caused an increase in IOP and led to a loss of axons, the hallmark of primary open-angle glaucoma. CCN2/CTGF appears to play a critical role in the homeostatic balance of the eye, so we investigated if CCN2/CTGF can modulate BMP and TGF-β signaling pathways in the outflow tissues. To this end, we analyzed the direct effect of CCN2/CTGF on both signaling pathways in two transgenic mouse models with a moderate (βB1-CTGF1) and a high CCN2/CTGF (βB1-CTGF6) overexpression and in immortalized human trabecular meshwork (HTM) cells. Additionally, we investigate whether CCN2/CTGF mediates TGF-β effects via different pathways. We observed developmental malformations in the ciliary body in βB1-CTGF6 caused by an inhibition of the BMP signaling pathway. In βB1-CTGF1, we detected a dysregulation of the BMP and TGF-β signaling pathways, with reduced BMP activity and increased TGF-β signaling. A direct CCN2/CTGF effect on BMP and TGF-β signaling was shown in immortalized HTM cells. Finally, CCN2/CTGF mediated its effects on TGF-β via the RhoA/ROCK and ERK signaling in immortalized HTM cells. We conclude that CCN2/CTGF functions as a modulator of the homeostatic balance of BMP and TGF-β signaling pathways, which is shifted in primary open-angle glaucoma.

## 1 Introduction

The multifunctional growth factor transforming growth factor (TGF)-β exists in three isoforms (TGF-β1, TGF-β2, TGF-β3). Among these, TGF-β2 is the predominant isoform in the eye and was detected in the aqueous humor, the vitreous, the neural retina and the retinal pigmented epithelium (Granstein et al., 1990; Jampel et al., 1990; Cousins et al., 1991; Pfeffer et al., 1994). The multiple functions of TGF-β2 are under tight control of a network of different growth factors and alterations of the homeostatic balance of growth factors can cause pathologic conditions (Fuchshofer and Tamm, 2012). It is suggested that such changes are partially responsible for the progress of ocular diseases like primary open-angle glaucoma (POAG), a neurodegenerative disease of the optic nerve (ON) and one of the leading causes of blindness in the western world (Quigley, 1996; Resnikoff et al., 2004). In the course of POAG, ON axons become damaged at the optic nerve head (ONH), leading to progressive loss of retinal ganglion cells (RGCs) and causing blindness in the end (Quigley et al., 1983). Multiple, randomized clinical studies revealed that intraocular pressure (IOP) is a major risk factor for axon loss in POAG and that lowering IOP reduces the progression of the disease (Collaborative Normal Tension Glaucoma Study, 1998a; Collaborative Normal Tension Glaucoma Study, 1998b; TheAGISInvestigators, 2000; Gordon et al., 2002; Leske et al., 2003). IOP is maintained by a balanced system of aqueous humor production and outflow. Aqueous humor is actively secreted by the ciliary epithelium of the ciliary body and is mainly drained via the conventional outflow pathway, consisting of the trabecular meshwork (TM) and Schlemm’s canal (SC) (Tamm, 2010; Tamm et al., 2015). IOP is generated by the resistance against aqueous humor outflow, provided by the juxtacanalicular tissue (JCT) of the TM and the endothelial cells of SC. Molecular effects leading to an increased resistance in aqueous humor outflow pathway and thereby creating an IOP, which is too high for the health of the eye, are not fully elucidated until know (Leske et al., 2003). However, it is known that the increased outflow resistance is caused by changes in the quantity and quality of extracellular matrix (ECM) and an increased actin-myosin contractility leading to a stiffening of the outflow tissues in POAG patients (Lutjen-Drecoll et al., 1989; Tian et al., 2000; Wiederholt et al., 2000; Hann et al., 2001; Tian et al., 2009). There are numerous indications that TGF-β2 is a key factor of the pathological changes by modulating synthesis and degradation of ECM in the TM (Fuchshofer and Tamm, 2012). Several studies could demonstrate higher amounts of TGF-β2 in aqueous humor samples of patients with POAG (Tripathi et al., 1994; Inatani et al., 2001; Picht et al., 2001; Ochiai and Ochiai, 2002; Ozcan et al., 2004; Yamamoto et al., 2005; Min et al., 2006; Trivedi et al., 2011). *In vitro* studies demonstrated that increased concentrations of TGF-β2 could cause the pathologic alterations observed in the outflow tissues of POAG patients and *in situ* and *in vivo* administration of active TGF-β2 led to an increase in outflow resistance (Fuchshofer and Tamm, 2012; Patil et al., 2022). Analysis of the molecular mechanisms in the outflow tissues revealed that the growth factor Cellular Communication Network Factor 2/Connective Tissue Growth Factor (CCN2/CTGF) acts downstream of TGF-β2 and mediates the changes in the ECM and the actin cytoskeleton (Grotendorst et al., 1996; Leask and Abraham, 2004; Junglas et al., 2009; Junglas et al., 2012; Overby et al., 2014). CCN2/CTGF is a member of the CCN family of matricellular regulatory proteins and is involved in various fibrotic diseases (Leask et al., 2002; Brigstock, 2003; Rachfal and Brigstock, 2005). In the human and mouse eye CCN2/CTGF is present in many different compartments and shows a high expression especially in the TM (Tomarev et al., 2003; van Setten et al., 2016; Dillinger et al., 2021). Increased concentrations of CCN2/CTGF are reported in the aqueous humor of various glaucoma forms (van Setten et al., 2002; Ho et al., 2005; Browne et al., 2011) and the lens specific overexpression of CCN2/CTGF causes elevated IOP and a decline of ON axons in transgenic mice (Junglas et al., 2012). CCN2/CTGF is structurally characterized by four conserved, cysteine-rich domains, which can coalesce interactions with other proteins. Members of the TGF-β-family like TGF-β1 and bone morphogenetic proteins (BMPs) can bind to the Willebrand type C repeats domain of CCN2/CTGF (Abreu et al., 2002) causing an increased TGF-β1 effect and diminished BMP signaling (Abreu et al., 2002). These findings indicate that CCN2/CTGF could function as a key modulator of the homeostatic balance between TGF-β2 and BMPs in the outflow tissues, as both factors modify each other’s signaling pathways in the TM (Wordinger et al., 2002; Fuchshofer et al., 2007; Fuchshofer et al., 2009). We hypothesize that CCN2/CTGF could shift the balance towards the pathogenesis of POAG. In this study, we investigate the effect of CCN2/CTGF on the BMP and the TGFβ signaling pathway *in vitro* and in the anterior eye segment, especially in the outflow tissues, using mouse models with a lens specific CCN2/CTGF overexpression to identify the underlying molecular mechanism of the pathogenesis of POAG.

## 2 Results

βB1-CTGF mouse lines were generated as described previously (Junglas et al., 2012). A total of six independent transgenic lines was generated. Two mouse lines, either with high transgenic expression, in the following referred to as βB1-CTGF6, or with moderate transgenic expression, referred to as βB1-CTGF1 (Junglas et al., 2012; Dillinger et al., 2022), were used in this study. βB1-CTGF6 mice showed a 3.8-fold higher *Ccn2*/*Ctgf* expression in the lens than βB1-CTGF1 mice (Supplementary Figure S1).

### 2.1 High overexpression of CCN2/CTGF in βb1-CTGF6 mice leads to developmental malformations of the ciliary body

**FIGURE 1:**
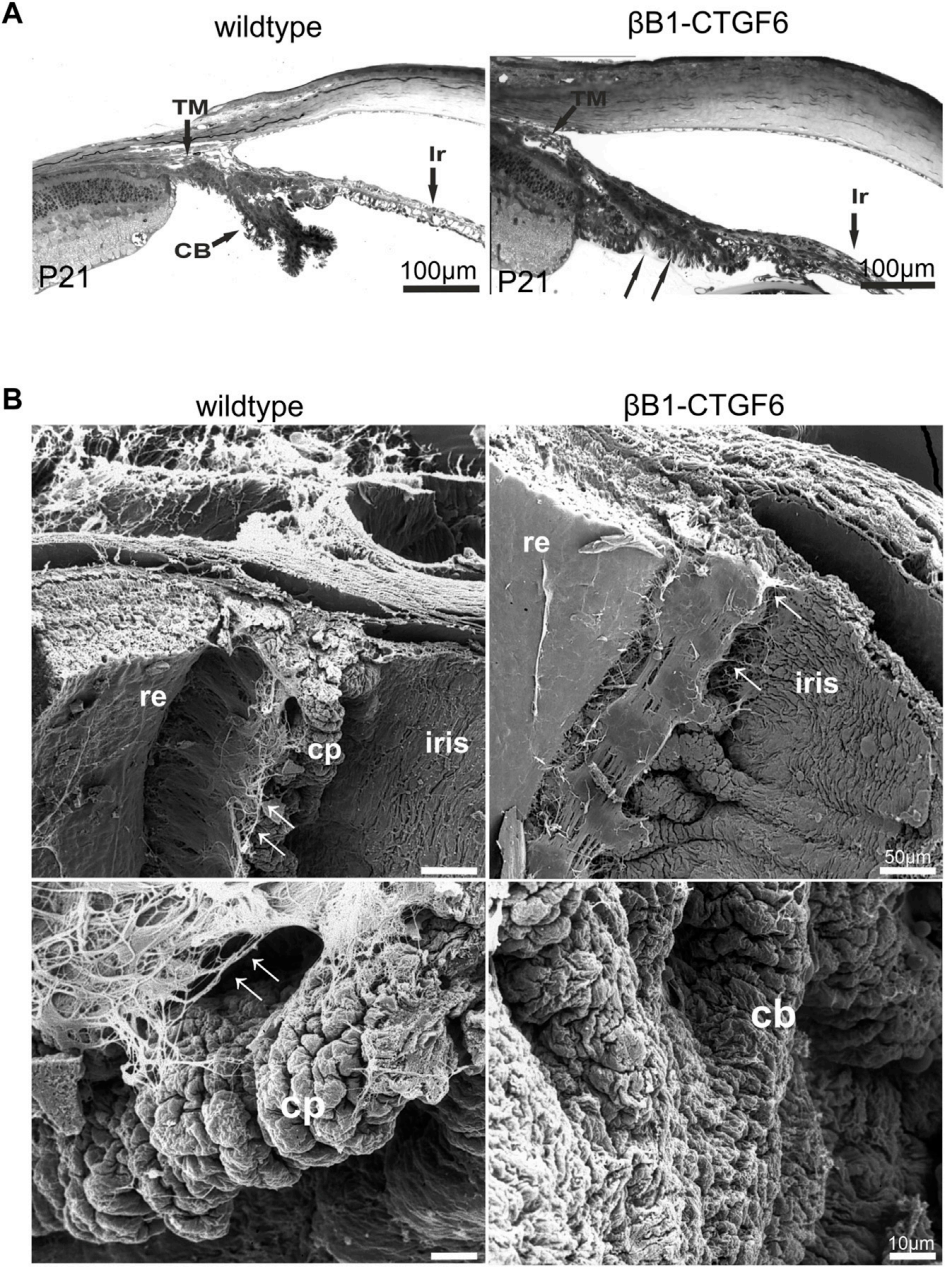
Ciliary body morphology of wildtype and βB1-CTGF6 mice. **(A)** Semithin sections of wildtype and βB1-CTGF6 mice at P21. At P21 the ciliary body is completely developed in wildtype mice. In contrast, in βB1-CTGF6 mice the defects in ciliary body development are indicated by the absence of ciliary processes (arrow). Ir, iris; CB, ciliary body; TM, trabecular meshwork; n = 3. **(B)** Scanning electron micrographs of the ciliary body of wildtype and βB1-CTGF6 mice at P21. Wildtype mice show a normal arrangement of ciliary processes and zonular fibers. In βB1-CTGF6 mice the number and length of folding of the ciliary epithelium is markedly decreased, accompanied with a reduction in the density of zonular fibers. cb, ciliary body; cp, ciliary processes; arrow: zonular fibers; n = 3.

The morphological analysis of the transgenic mice of the βB1-CTGF1 line with a moderate expression of CCN2/CTGF was already described in a previous publication showing no signs of developmental changes in the anterior chamber angle (Junglas et al., 2012), whereas surprisingly the transgenic mouse line with a high expression of CCN2/CTGF (βB1-CTGF6) showed distinct developmental abnormalities in the anterior eye segment. At postnatal day (P) 21, the ciliary body and processes were fully developed in wildtype mice and showed their typical structure with radial folds of the inner and outer ciliary epithelium, whereas in the βB1-CTGF6 mice only a slight folding of the ciliary epithelium could be observed (Figure 1A, arrow). In adult βB1-CTGF6 transgenic mice (P21) the ciliary processes of the ciliary body were not formed and in semithin sections it was difficult to distinguish between the region of ciliary processes and the iris root. Beside the changes of the ciliary body, hypoplasia of the iris was observed. To investigate whether the development of ciliary processes was affected in the entire circumference of the eye, we performed scanning electron microscopy of the ciliary body of wildtype and βB1-CTGF6 mice at P21 (Figure 1B), as the histological analysis in semithin sections is limited in showing the three-dimensional structure of the ciliary processes. At this stage of ciliary body development, wildtype mice showed a normal organization of ciliary processes, with the typical irregular pattern (Napier and Kidson, 2005). Zonular fibers, spanning between the ciliary body and the lens, are developed normally. In contrast, dramatic malformations were observed in age-matched βB1-CTGF6 mice. The ciliary body lacked the exact arrangement, with a reduction in length and folding of the ciliary processes, which was accompanied by a decrease in zonular fiber density (Figure 1B, arrow).

**FIGURE 2:**
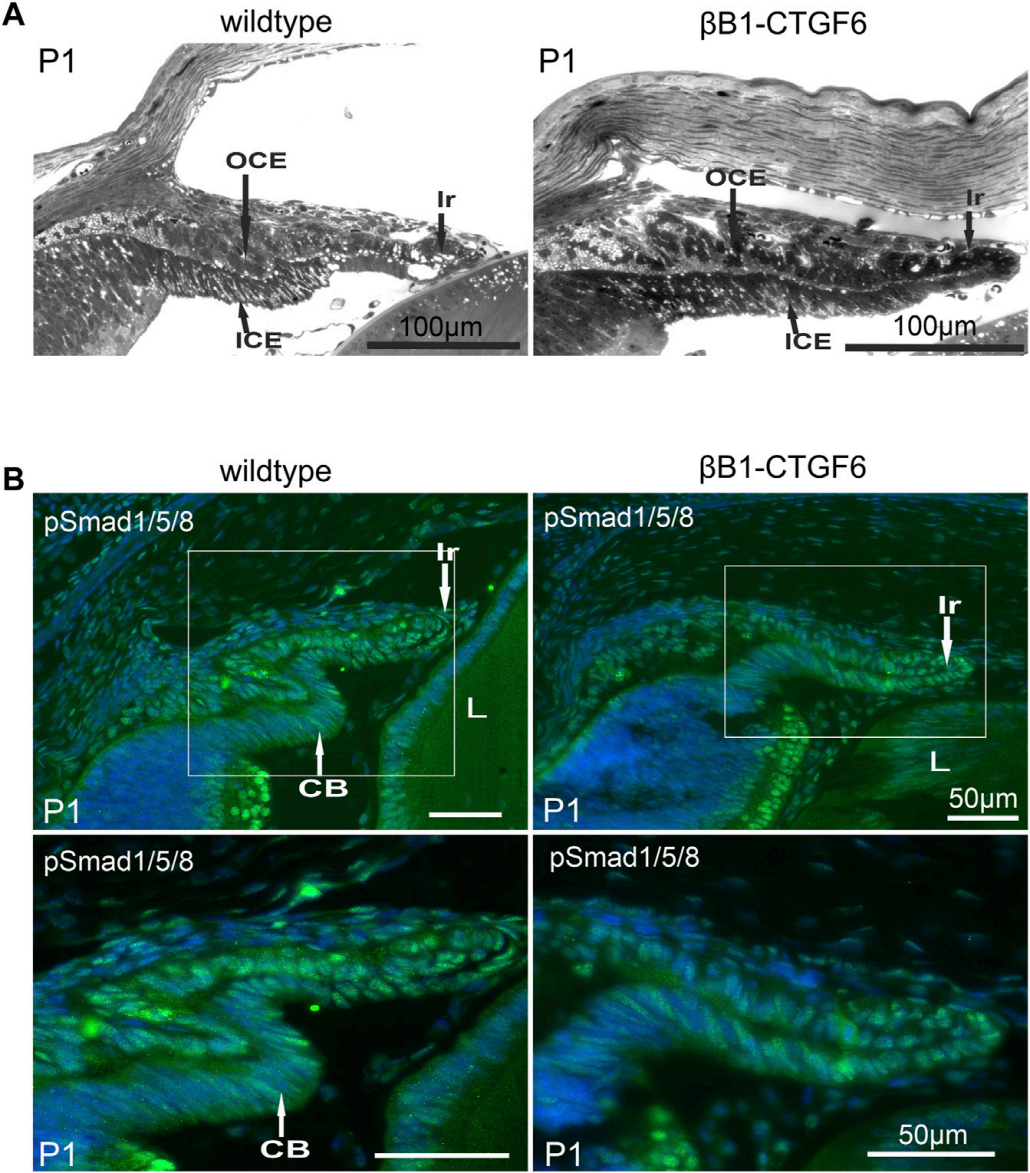
Ciliary body morphology and pSmad1/5/8 levels on P1 of wildtype and βB1-CTGF6 mice. **(A)** Semithin sections of wildtype and βB1-CTGF6 mice at P1. n = 3 **(B)** Immunohistochemical staining of pSmad1/5/8 (green) in the ciliary body of wildtype and βB1-CTGF6 mice at P1. Fluorescence intensity of pSmad1/5/8 (green) was markedly decreased in the ciliary epithelium of transgenic βB1-CTGF6 mice, compared to age-matched wildtype littermates. Nuclei were stained with Dapi (blue). OCE: outer ciliary epithelium; ICE: inner ciliary epithelium; Ir: Iris; L: lens; n = 5.

The formation of ciliary processes only occurs postnatally, and from P0 to P2 distinct structural changes within the ciliary epithelia occur, resulting in the formation of the ciliary processes (Napier and Kidson, 2005). We chose P1 to analyze whether the malformation of ciliary processes could already be observed. At P1 columnar cells of the outer ciliary epithelium could be distinguished from the cuboidal cells of the retinal pigment epithelium in wildtype animals (Figure 2A). The forming processes appeared to develop from an initial bulging of the outer ciliary epithelium inward toward the inner ciliary epithelium (Figure 2A) and a sharp separation between iris and ciliary body can be seen. The transgenic βB1-CTGF6 showed defects in the folding of inner ciliary epithelium and distinction between the epithelium of the iris and the inner ciliary epithelium was not possible. The elongation of the iris was reduced (Figure 2A). These new findings led us to a re-evaluation of our previous statement, that the βB1-CTGF1 line with a moderate CCN2/CTGF overexpression shows no obvious developmental abnormalities within the eye. Therefore, we performed detailed analysis of the ciliary body of the βB1-CTGF1 line in comparison to wildtype littermates, to examine whether a moderate CCN2/CTGF expression would also lead to developmental malformations in the ciliary body. We performed immunohistochemical staining against Cluster of differentiation 31 (CD31), a marker for endothelial cells, on whole mounts of the ciliary body of wildtype and βB1-CTGF1 mice at the age of 4 weeks. *En face* imaging of the ciliary body showed CD31 positives endothelial cells forming the vascular plexus in the ciliary processes of wildtype littermates. In comparison to the vascular plexus of ciliary processes from wildtype mice, βB1-CTGF1 mice showed no differences in the formation the ciliary body vasculature (Supplementary Figure S2).

The altered morphology of the ciliary processes and iris in the βB1-CTGF6 was comparable to the developmental changes described in a transgenic mouse line with a lens specific Noggin expression (Zhao et al., 2002), where the absence of ciliary body formation was shown to be due to the inhibition of BMP signaling. Staining against phosphorylated Smad1/5/8 (pSmad1/5/8) confirmed an active BMP signaling pathway in both epithelial layers of the ciliary body and in the iris of wildtype mice (Figure 2B). The strongest signal was observed in the outer ciliary epithelium and in the iris stroma. Signal for pSmad1/5/8 was markedly reduced in the βB1-CTGF6 line in the ciliary body and in the iris (Figure 2B), indicating an impaired BMP signaling in the anterior eye segment of the transgenic mice.

Since the lens of the βB1-CTGF6 line develops cataract and bursts at the posterior pole on P21, further analysis of the eyes after P21 was not possible. Hence, we switched to the initial transgenic βB1-CTGF1 line to study the effects of CCN2/CTGF on BMP and TGF-β signaling.

### 2.2 BMP signaling is inhibited in transgenic βb1-CTGF1 mice

**FIGURE 3:**
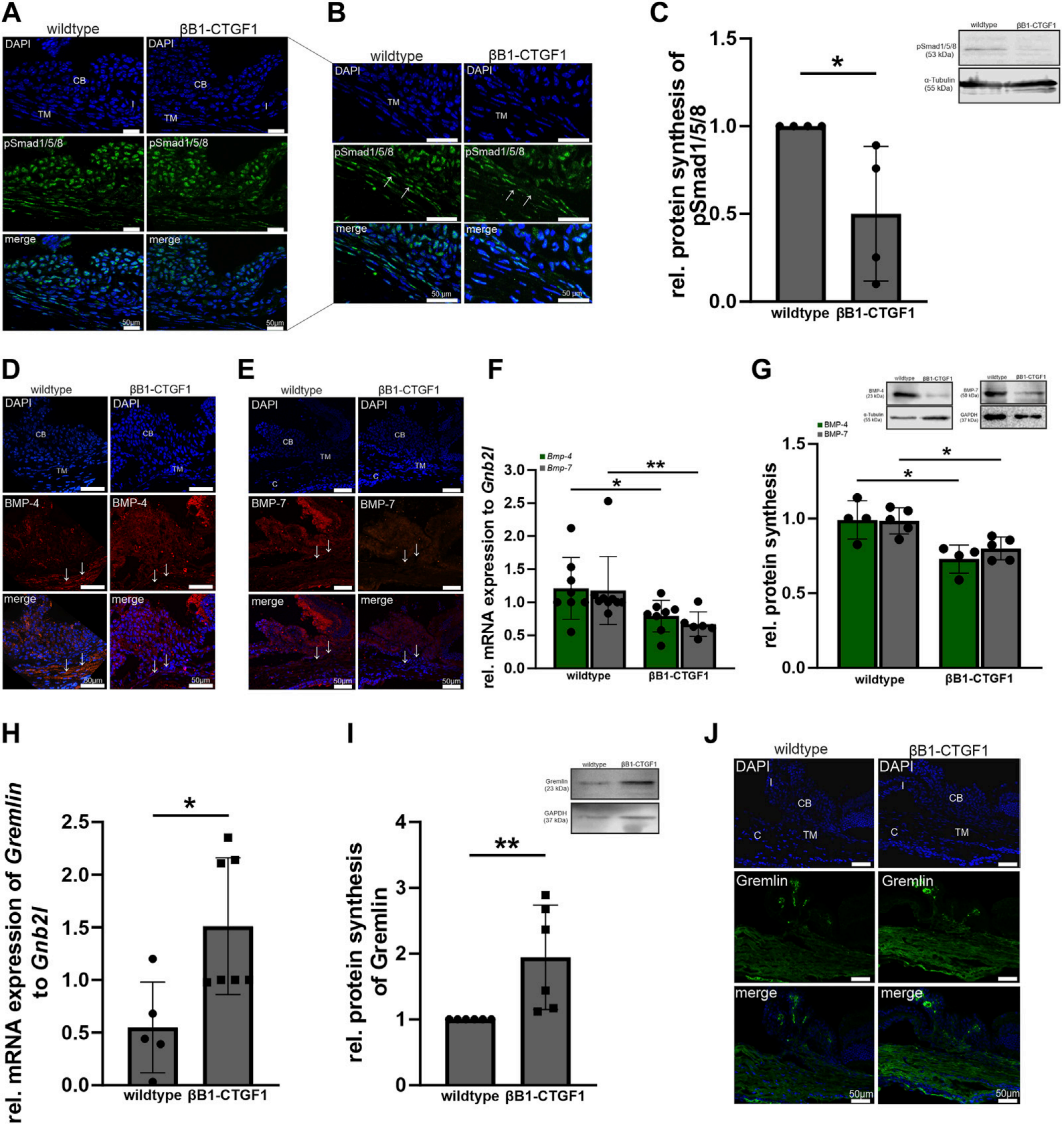
BMP signaling in the anterior eye segment of βB1-CTGF1 mice. **(A, B)** pSmad1/5/8 immunoreactivity (green) in the anterior chamber angle of 2-month-old βB1-CTGF1 mice and wildtype littermates. Immunoreactivity of pSmad1/5/8 was reduced in the TM of transgenic mice, compared to wildtype mice. Nuclei were stained with Dapi (blue); n = 5. **(C)** Western blot analysis revealed a significantly reduced phosphorylation of pSmad1/5/8 in the anterior eye segment of βB1-CTGF1 mice, compared to wildtype littermates (n = 4) Mean value of wildtype animals (control) was set at 1. Total protein stained with Coomassie was used to normalize protein synthesis. Data represented as mean ± SD. Right panel shows a representative Western blot. For statistical analysis the Mann-Whitney test was used. **(D, E)** BMP-4 and BMP-7 immunoreactivity (red) in the anterior chamber angle of 2-month-old βB1-CTGF1 mice and wildtype littermates. Immunoreactivity of BMP-4 and BMP-7 was dramatically reduced in the TM of transgenic animals, compared to control animals. Nuclei were stained with Dapi; n = 5 each. **(F)** Real-time RT-PCR analysis of *Bmp-4* and *Bmp-7* in the anterior eye segment of 2-month-old βB1-CTGF1 mice and wildtype littermates. mRNA expression of *Bmp-4* and *Bmp-7* was significantly reduced in βB1-CTGF1 mice compared to wildtype mice (*Bmp-4*: WT: n = 8, TG: n = 8; *Bmp-7*: WT: n = 9, TG: n = 6). mRNA expression was normalized to *Gnb2l,* and mean value of wildtype mice was set to 1. For statistical analysis the Mann-Whitney test was used. **(G)** Western blot analyses of BMP-4 and BMP-7 in the anterior eye segment of 2-month-old βB1-CTGF1 mice and wildtype littermates. Protein synthesis of BMP-4 and BMP-7 was significantly reduced in transgenic animals compared to wildtype controls (BMP-4: n = 4 each; BMP-7: n = 5 each). Integrated Blots in the graph show a representative Western blot for both proteins. For statistical analysis the Mann-Whitney test was used. **(H)** Real-time RT-PCR analyses of *Gremlin* in the anterior eye segment of 2-month-old βB1-CTGF1 mice and wildtype littermates. mRNA expression of *Gremlin* was dramatically increased in βB1-CTGF1 mice, compared to wildtype littermates (WT: n = 5, TG: n = 7). mRNA expression was normalized to *Gnb2l* and mean value of wildtype mice was set to 1. For statistical analysis the Mann-Whitney test was used. **(I)** Western blot analysis of *Gremlin* in the anterior eye segment of 2-month-old βB1-CTGF1 mice and wildtype littermates. Protein synthesis was significantly increased in βB1-CTGF1 mice, compared to wildtype (n = 6). For statistical analysis the Mann-Whitney test was used. **(J)** Immunoreactivity of Gremlin (green) was increased in the TM of 2-month-old βB1-CTGF mice, compared to wildtype control littermates. Nuclei were stained with Dapi (blue). CB: ciliary body; I, iris; TM: trabecular meshwork; c, cornea; n = 5. *p ≤ 0.05; **p < 0.01.

The βB1-CTGF1 mice does not show any signs of developmental malformation but develops progressively increasing intraocular pressure and a progressive loss of ON axons over time (Junglas et al., 2012; Dillinger et al., 2022). Therefore, we used this mouse line to investigate, whether the BMP and TGF-β signaling pathways are altered by the moderate overexpression of CCN2/CTGF. Previous *in vitro* studies have shown that the interplay between BMPs and TGF-β might play a role in the ECM homeostasis of the trabecular meshwork, causing increased outflow resistance (Fuchshofer et al., 2007; Wordinger et al., 2007). Primarily, we verified active BMP signaling in the outflow tissues. Therefore, we stained cross sections of the anterior eye segment of 2-month-old βB1-CTGF1 and wildtype mice against pSmad1/5/8 (Figures 3A, B). We could identify a fluorescence signal in the iris, ciliary body and the TM (Figure 3B, arrow) of wildtype mice, this signal was slightly decreased solely in the TM in βB1-CTGF1 littermates (Figure 3B, arrow). This observation could be confirmed by Western Blot analysis of anterior eye segments, including the cornea, iris, ciliary body and TM of 2-month-old βB1-CTGF1 and wildtype mice. pSmad1/5/8 protein synthesis was significantly decreased in βB1-CTGF1 mice (0.50 ± 0.38), compared to wildtype littermates (Figure 3C). The quantification of pSmad1/5/8 positive fluorescence area in the TM could show a significant decrease in βB1-CTGF1 mice, compared to wildtype littermates (Supplementary Figure S3A; WT: 1.24 ± 0.38, βB1-CTGF1: 0.46 ± 0.24). Next, we were interested whether BMP ligands are altered in our transgenic glaucoma mouse model and thereby lead to the reduced BMP signaling activity. Therefore, we performed immunohistochemical staining, real-time RT-PCR and Western blot analysis of BMP-4 and BMP-7 in anterior eye segments. BMP-4 could be detected in the ciliary body and more pronouncedly in the TM (Figure 3D, arrow) in 2-month-old wildtype mice (Figure 3D). In contrast, in 2-month-old βB1-CTGF1 mice, the signal for BMP-4 was dramatically reduced in the TM (Figure 3D, arrow) and remains unaffected in the ciliary body of the transgenic animals (Figure 3D). The quantification of BMP-4 positive fluorescence area in the TM could show a significant decrease in βB1-CTGF1 mice, compared to wildtype littermates (Supplementary Figure S3B; WT: 0.99 ± 0.18, βB1-CTGF1: 0.20 ± 0.10). In comparison to the localization of BMP-4, BMP-7 signal was mostly present in the ciliary body of 2-month-old wildtype mice and showed only weak immunoreactivity in the TM (Figure 3F, arrow. However, in the transgenic βB1-CTGF1 mice, the signal for BMP-7 was completely absent in the TM (Figure 3E, arrow) and was markedly reduced in the ciliary body (Figure 3E). The immunohistochemical observation could be confirmed by real-time RT-PCR and Western blot analysis of BMP-4 and BMP-7 in anterior eye segments of 2-month-old βB1-CTGF1 and wildtype mice. mRNA expression of *Bmp-4* and *Bmp-7* was significantly decreased in anterior eye segment samples of βB1-CTGF1 mice (*Bmp-4*: 0.65 ± 0.24; *Bmp-7*: 0.57 ± 0.19), compared to their wildtype littermates (*Bmp-4*: 1.00 ± 0.47; *Bmp-7*: 1.00 ± 0.51; Figure 3F). The BMP-4 and BMP-7 protein synthesis was significantly decreased in βB1-CTGF1 mice (BMP-4: WT: 0.99 ± 0.13, βB1-CTGF1: 0.73 ± 0.09; BMP-7: WT: 0.98 ± 0.09, βB1-CTGF1: 0.80 ± 0.08), compared to wildtype control mice (Figure 3G). Finally, we analyzed the specific BMP inhibitor Gremlin in anterior eye segments of 2-month-old βB1-CTGF1 and wildtype mice (Figures 2H–J). Immunofluorescence staining against Gremlin showed a localization in the TM and the ciliary body in wildtype animals. This signal was more intense in the TM and the ciliary body in βB1-CTGF1 mice, compared to control littermates (Figure 3J). This observation could be verified by real-time RT-PCR and Western blot analysis. mRNA expression of *Gremlin* was significantly increased in βB1-CTGF1 mice (2.76 ± 0.65), compared to wildtype littermates (1.00 ± 0.43; Figure 3H). Also, the protein synthesis of Gremlin was significantly increased in βB1-CTGF1 mice (1.94 ± 0.79), compared to wildtype control mice (Figure 3I).

### 2.3 CCN2/CTGF attenuates BMP signaling in HTM-N cells *in vitro*

**FIGURE 4:**
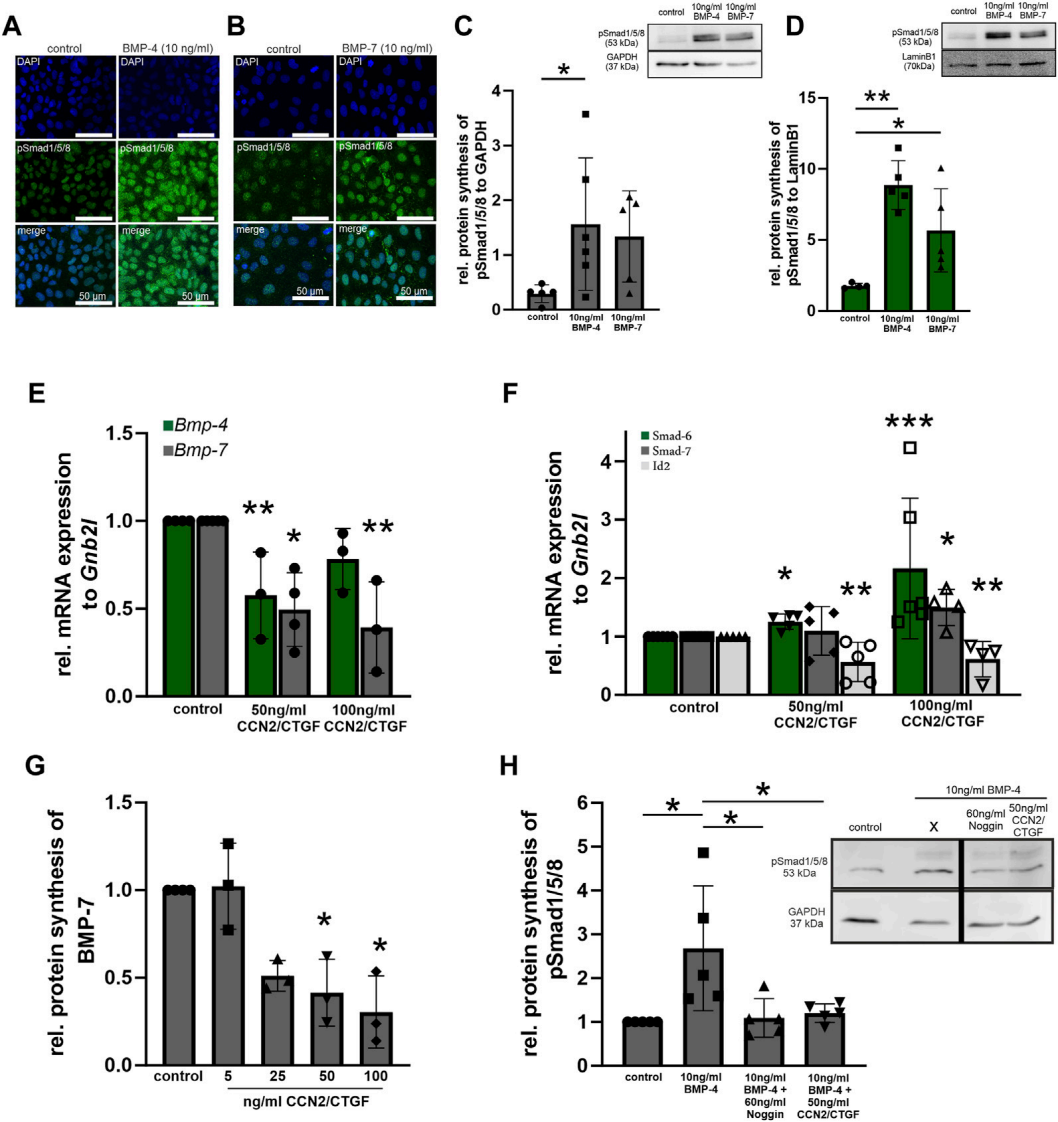
BMP signaling in CCN2/CTGF treated HTM-N cells *in vitro*. **(A, B)** Verification of BMP signaling activity in HMT-N cells *in vitro*. Immunoreactivity of pSmad1/5/8 (green) in HMT-N cells was increased after the treatment with 10 ng/mL BMP-4 **(A)** and 10 ng/mL BMP-7 **(B)**. Nuclei were stained with Dapi (blue). n = 3 **(C)** Western blot analysis of pSmad1/5/8 in the cytoplasmic fraction of HTM-N cells after the treatment with 10 ng/mL BMP-4 or BMP-7 for 1 h. Protein synthesis of pSmad1/5/8 was significantly increased after the treatment with BMP-4 for. (n = 5). GAPDH was used to normalize protein synthesis. Data represented as mean ± SD. Right panel shows a representative Western blot. For statistical analysis unpaired two-tailed *t*-test was used. **(D)** Western blot analysis of pSmad1/5/8 in the nuclear fraction of HTM-N cells after the treatment with 10 ng/mL BMP-4 or BMP-7 for 1 h. Protein synthesis of pSmad1/5/8 was significantly increased after 1 h with both treatments (n = 5). LaminB1 was used to normalize protein synthesis. Data represented as mean ± SD. Right panel shows a representative Western blot. For statistical analysis unpaired two-tailed *t*-test was used. **(E)** Real-time RT-PCR analysis of *Bmp-4* and *Bmp-7* after the treatment with 50 ng/mL and 100 ng/mL CCN2/CTGF for 24 h in HMT-N cells. mRNA expression of *Bmp-4* and *Bmp-7* was significantly reduced after the treatment with CCN2/CTGF (*Bmp-4*: control n = 4, 50 ng/mL CCN2/CTGF n = 3, 100 ng/mL CCN2/CTGF n = 3; *Bmp-7*: control n = 5, 50 ng/mL CCN2/CTGF n = 4, 100 ng/mL CCN2/CTGF n = 3). mRNA expression was normalized to *Gnb2l,* and mean value of untreated control cells was set to 1. For statistical analysis the Kruskal–Wallis test was used. **(F)** Real-time RT-PCR analyses of *Smad6*, *Smad7*, and *Id2* after the treatment with 50 ng/mL and 100 ng/mL CCN2/CTGF for 24 h in HMT-N cells. mRNA expression of *Smad6* was significantly increased after the treatment with 100 ng/mL CCN2/CTGF (n = 6). mRNA expression of Smad7 was significantly increased after the treatment with 100 ng/mL CCN2/CTGF (control: n = 5, 50 ng/mL CCN2/CTGF: n = 5, 100 ng/mL CCN2/CTGF: n = 4). mRNA expression of *Id2* was significantly reduced after the treatment with 50 ng/mL and 100 ng/mL CCN2/CTGF (control: n = 5, 50 ng/mL CCN2/CTGF: n = 5, 100 ng/mL CCN2/CTGF: n = 4). mRNA expression was normalized to *Gnb2l,* and mean value of untreated control cells was set to 1. For statistical analysis the Kruskal–Wallis test was used. **(G)** Western blot analyses of BMP-7 after the treatment with 5 ng/mL, 25 ng/mL, 50 ng/mL and 100 ng/mL CCN2/CTGF for 24 h in HMT-N cells. Protein synthesis of BMP-7 was significantly reduced after the treatment with 50 ng/mL and 100 ng/mL CCN2/CTGF (control: n = 4, 5 ng/mL CCN2/CTGF: n = 3, 25 ng/mL CCN2/CTGF: n = 3, 50 ng/mL CCN2/CTGF: n = 3, 100 ng/mL CCN2/CTGF: n = 3). Mean value of wildtype animals (control) was set to 1. *α*-Tubulin was used to normalize protein synthesis. For statistical analysis the Kruskal–Wallis test was used. **(H)** Western blot analysis of pSmad1/5/8 after the treatment with 10 ng/mL BMP-4, 60 ng/mL Noggin and 10 ng/mL BMP-4, and 50 ng/mL CCN2/CTGF and 10 ng/mL BMP-4 in HTM-N cells. pSmad1/5/8 protein synthesis was increased after the treatment with BMP-4 (n = 5), compared to untreated control cells and significantly reduced after the treatment with the combination of Noggin and BMP-4 (n = 5) and the combination of CCN2/CTGF and BMP-4 (n = 5), compared to the treatment with BMP-4 only. Mean value of wildtype animals (control) was set to 1. GAPDH was used to normalize protein synthesis. Data represented as mean ± SD. For statistical analysis the One-way ANOVA test was used. *p ≤ 0.05, **p < 0.01, ***p < 0.001.

To investigate whether the observed effect in the transgenic animals *in vivo* is a direct effect of CCN2/CTGF on the BMP signaling pathway, we choose HTM-N cells to further analyze the direct potential of CCN2/CTGF to influence the BMP signaling activity *in vitro* (Figure 4). Before we analyzed the effect of CCN2/CTGF on BMP signaling in HTM-N cells, we confirmed active BMP signaling in this cell line (Figures 4A–C). HTM-N cells were treated with 10 ng/mL BMP-4 or BMP-7 for 1, 3, 6 or 24 h. Immunocytochemical staining showed a fluorescence signal of pSmad1/5/8 localized in the cell nucleus, which was strongly enhanced in HTM-N cells after BMP-4 or BMP-7 treatment for 1 h, compared to untreated control cells (Figures 4A, B). To analyze the induction of BMP signaling in HTM-N cells after BMP-4 and BMP-7 treatment more precisely, we isolated the cytoplasmic and nuclear protein fraction from BMP-4 and BMP-7 treated HTM-N cells and performed a Western blot against pSmad1/5/8 (Figures 4C, D). In the cytoplasmic fraction pSmad1/5/8 protein level was significantly elevated after the treatment with 10 ng/mL BMP-4 (control: 0.29 ± 0.16; BMP-4: 1.56 ± 1.21; BMP-7: 1.34 ± 0.83; Figure 4C). This effect was even more pronounced in the nuclear protein fraction, in which pSmad1/5/8 protein level was significantly elevated after the treatment with 10 ng/mL BMP-4 or 10 ng/mL BMP-7 (control: 1.76 ± 0.18; BMP-4: 8.86 ± 1.71; BMP-7: 5.67 ± 2.93, Figure 4D). Next, we wanted to investigate if CCN2/CTGF can inhibit the BMP signaling pathway. Therefore, HTM-N cells were treated with 50 ng/mL or 100 ng/mL CCN2/CTGF or left untreated as a control. We performed real-time RT-PCR analysis for *Bmp-4*, *Bmp-7*, *Smad6*, *Smad7* and *Id2*. mRNA expression of *Bmp-4* was significantly decreased after the treatment with 50 ng/mL and 100 ng/mL CCN2/CTGF compared to untreated HTM-N cells (50 ng/mL CCN2/CTGF: 0.58 ± 0.5; 100 ng/mL CCN2/CTGF: 0.78 ± 0.17; Figure 4E). The effect of CCN2/CTGF treatment on *Bmp-7* mRNA levels was more pronounced. *Bmp7* mRNA expression was significantly decreased after the treatment with 50 ng/mL and 100 ng/mL CCN2/CTGF, compared to untreated HTM-N cells (50 ng/mL CCN2/CTGF: 0.5 ± 0.21; 100 ng/mL CCN2/CTGF: 0.39 ± 0.26; Figure 4E). Further, we analyzed the mRNA expression of inhibitory Smad proteins of the BMP signaling pathway. *Smad6* mRNA levels were significantly increased after the treatment with 50 ng/mL CCN2/CTGF or 100 ng/mL CCN2/CTGF compared to controls (50 ng/mL CCN2/CTGF: 1.13 ± 0.35; 100 ng/mL CCN2/CTGF: 2.17 ± 1.28; Figure 4F). The mRNA expression of *Smad7*, which can act on the BMP and the TGF-β pathway, was significantly increased after the treatment with 100 ng/mL CCN2/CTGF compared to untreated controls (50 ng/mL CCN2/CTGF: 1.10 ± 0.42; 100 ng/mL CCN2/CTGF: 1.50 ± 0.31; Figure 4F). Finally, we analyzed the early response gene of the BMP signaling pathway, DNA-binding protein inhibitor (ID2). The mRNA expression of *Id2* was significantly decreased after the treatment with 50 ng/mL and 100 ng/mL CCN2/CTGF compared to controls (50 ng/mL CCN2/CTGF: 0.56 ± 0.34; 100 ng/mL CCN2/CTGF: 0.61 ± 0.30; Figure 4F). The inhibitory effect of CCN2/CTGF on the BMP signaling in HTM-N cells could be confirmed by Western blot analysis of BMP-7 (Figure 4G). BMP-7 protein synthesis was significantly decreased after the treatment with 50 ng/mL or 100 ng/mL CCN2/CTGF compared to controls (5 ng/mL CCN2/CTGF: 1.02 ± 0.25; 25 ng/mL CCN2/CTGF: 0.51 ± 0.09; 50 ng/mL CCN2/CTGF: 0.41 ± 0.19; 100 ng/mL CCN2/CTGF: 0.30 ± 0.21; Figure 4G). Finally, we proved the inhibitory effect of CCN2/CTGF on the BMP signaling pathway in HTM-N cells by comparing its potential to the effect of Noggin, a specific BMP inhibitor (Figure 4H). HTM-N cells were treated either with 10 ng/mL BMP-4 alone, or a combination of 60 ng/mL Noggin and 10 ng/mL BMP-4 or with the combination of 50 ng/mL CCN2/CTGF and 10 ng/mL BMP-4. BMP-4 treatment alone increased pSmad1/5/8 protein synthesis in HTM-N cells after 24 h (2.68 ± 1.42). The treatment with Noggin in combination with BMP-4 inhibited the BMP-4 induced increased of pSmad1/5/8 (1.09 ± 0.44), compared to control cells. Interestingly, CCN2/CTGF treatment in combination with BMP-4 showed a similar effect and inhibited the activation of BMP-4 signaling (1.2 ± 0.21), indicating the potent effect of CCN2/CTGF as an inhibitor of the BMP signaling pathway (Figure 4H).

### 2.4 TGF-β signaling is increased by CCN2/CTGF in transgenic βb1-CTGF1 mice *in vivo* and in HTM-N cells *in vitro*

**FIGURE 5:**
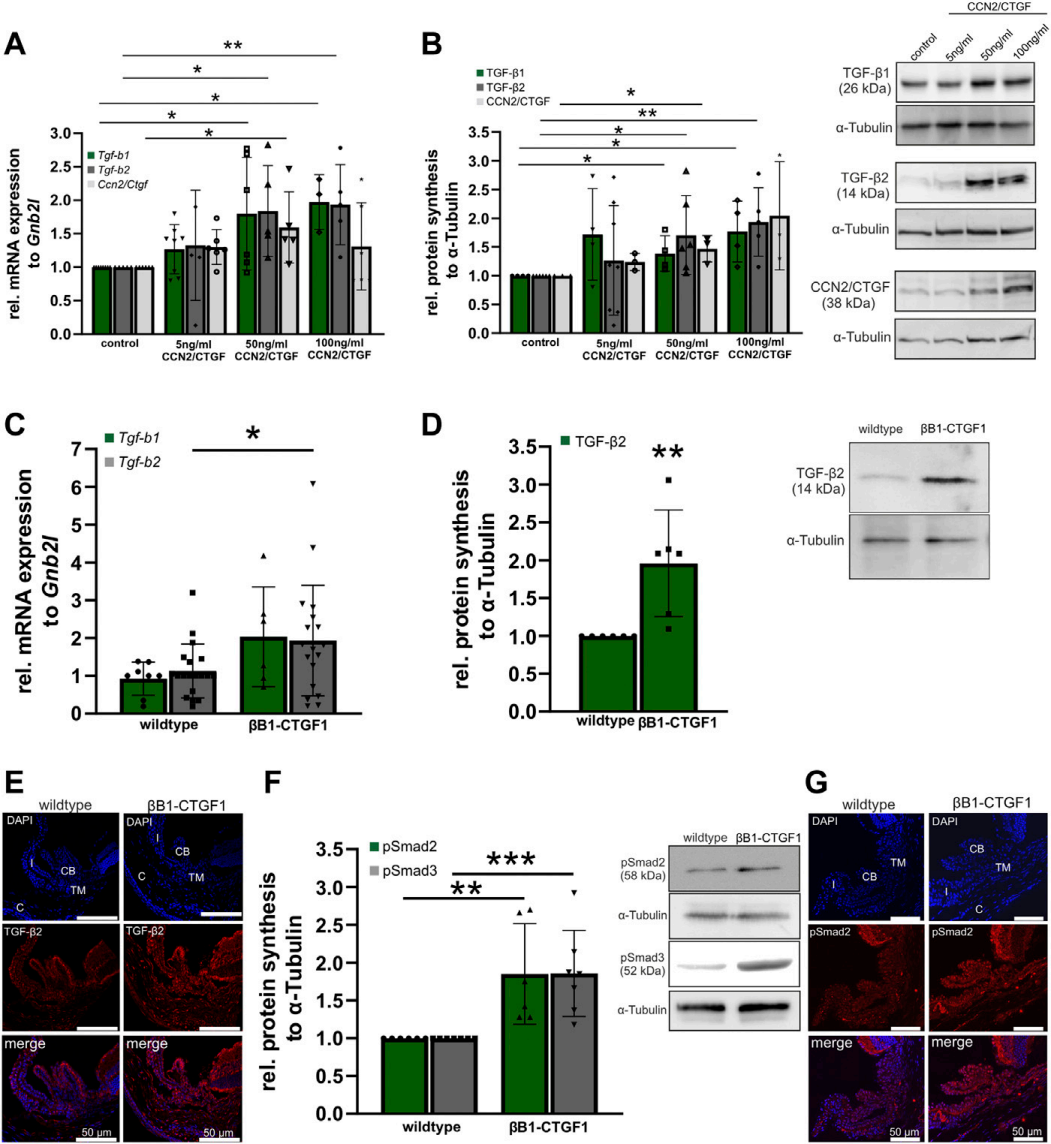
TGF-β signaling in CCN2/CTGF treated HTM-N cells *in vitro* and in βB1-CTGF1 mice *in vivo*. **(A)** Real-time RT-PCR analyses of *Tgf-b1*, *Tgf-b2* and *Ccn2/Ctgf* in HTM-N cells after the treatment with 5 ng/mL, 50 ng/mL and 100 ng/mL CCN2/CTGF for 24 h. mRNA expression of *Tgf-b1* was significantly increased after the treatment with 50 ng/mL and 100 ng/mL CCN2/CTGF (control: n = 8, 5 ng/mL CCN2/CTGF: n = 8, 50 ng/mL CCN2/CTGF: n = 6, 100 ng/mL CCN2/CTGF: n = 3). *Tgf-b2* mRNA was significantly enhanced after the treatment with 50 ng/mL and 100 ng/mL CCN2/CTGF (control: n = 5, 5 ng/mL CCN2/CTGF: n = 5, 50 ng/mL CCN2/CTGF: n = 5, 100 ng/mL CCN2/CTGF: n = 5). mRNA expression of *Ccn2/Ctgf* was significantly increased after the treatment with 5 ng/mL and 50 ng/mL CCN2/CTGF (control: n = 6, 5 ng/mL CCN2/CTGF: n = 6, 50 ng/mL CCN2/CTGF: n = 5, 100 ng/mL CCN2/CTGF: n = 6). mRNA expression was normalized to *Gnb2l,* and mean value of untreated control cells was set to 1. For statistical analysis the Kruskal–Wallis test was used. **(B)** Western blot analysis of TGF-β1 and TGF-β2 in HTM-N cells after the treatment with 5 ng/mL, 50 ng/mL and 100 ng/mL CCN2/CTGF for 24 h. Protein synthesis of TGF-β1 was significantly increased after the treatment with 50 ng/mL and 100 ng/mL CCN2/CTGF (n = 4). The protein synthesis of TGF-β2 was significantly enhanced after the treatment with 50 ng/mL and 100 ng/mL CCN2/CTGF (control: n = 6, 5 ng/mL CCN2/CTGF: n = 8, 50 ng/mL CCN2/CTGF: n = 6, 100 ng/mL CCN2/CTGF: n = 5). Right panel shows representative Western Blots for the proteins. Mean value of untreated control cells was set at 1. *α*-Tubulin was used to normalize protein synthesis. For statistical analysis the Kruskal–Wallis test was used. **(C)** Real-time RT-PCR analysis of *Tgf-b1*and *Tgf-b2* in the anterior eye segment of 2-month-old βB1-CTGF1 mice and wildtype littermates. mRNA expression of *Tgf-b2* was significantly increased in βB1-CTGF mice, compared to wildtype littermates (*Tgf-b1*: WT: n = 8, TG: n = 6; *Tgf-b2*: WT: n = 19, TG: n = 19). For statistical analysis the Mann-Whitney test was used. mRNA expression was normalized to *Gnb2l,* and mean value of untreated control cells was set to 1. **(D)** Western blot analysis of TGF-β2 in the anterior eye segment of 2-month-old βB1-CTGF mice and wildtype littermates. Protein synthesis of TGF-β2 was significantly increased in βB1-CTGF1 mice, compared to wildtype littermates (TGF-β2: WT: n = 6, TG: n = 6). Right panel shows a representative Western blot. *α*-Tubulin was used to normalize protein synthesis. For statistical analysis the Mann-Whitney test was used. **(E)** Immunohistochemical staining of TGF-β2 (red) in the anterior chamber of 2-month-old βB1-CTGF1 mice and wildtype littermates. Immunoreactivity of TGF-β2 was increased in the TM of βB1-CTGF mice1, in comparison to wildtype mice. Nuclei were stained with Dapi (blue). n = 5 **(F)** Western blot analysis of the phosphorylation of Smad2 and Smad3. Protein synthesis of pSmad2 and pSmad3 was significantly increased in βB1-CTGF mice, compared to wildtype littermates (pSMAD2: WT: n = 6, TG: n = 6; pSmad3: WT: n = 7, TG: n = 7). Right panel shows representative Western Blots for both proteins. *α*-Tubulin was used to normalize protein synthesis. For statistical analysis the Mann-Whitney test was used. **(G)** Immunohistochemical staining of pSmad2 (red) in the anterior chamber angle of 2-month-old βB1-CTGF1 mice and wildtype littermates. Immunoreactivity of pSmad2 is increased in the TM of βB1-CTGF1 mice, in comparison to wildtype mice. Nuclei were stained with Dapi (blue). Data represented as mean ± SD. CB: ciliary body, I: iris, C: cornea, TM: trabecular meshwork. n = 5. *p ≤ 0.05, **p < 0.01, ***p < 0.001.

It is already known that CCN2/CTGF is a downstream mediator of TGF-βs (Fuchshofer et al., 2005), but little is known about the effect of CCN2/CTGF on TGF-β signaling. To learn more about the relationship of CCN2/CTGF and TGF-β signaling we first analyzed the effect of CCN2/CTGF treatment in HTM-N cells on the expression and protein synthesis of TGF-β1, TGF-β2, and CCN2/CTGF itself *in vitro* (Figure 5). mRNA expression of *Tgf-b1* was significantly increased after the treatment with 50 ng/mL and 100 ng/mL CCN2/CTGF (5 ng/mL: 1.27 ± 0.37; 50 ng/mL: 1.8 ± 0.84; 100 ng/mL: 1.97 ± 0.41), compared to untreated control cells (Figure 4A). Similar effects could be seen for the expression of *Tgf-b2*, whose mRNA expression was significantly increased after the treatment with 50 ng/mL and 100 ng/mL CCN2/CTGF (5 ng/mL 1.33 ± 0.82; 50 ng/mL: 1.84 ± 0.68; 100 ng/mL: 1.93 ± 0.60), compared to untreated control cells (Figure 5A). Interestingly, CCN2/CTGF treatment in HTM-N cells showed an auto-inductive effect on *Ccn2/Ctgf* mRNA expression. *Ccn2/Ctgf* mRNA levels were significantly increased after the treatment with 50 ng/mL CCN2/CTGF (5 ng/mL: 1.3 ± 0.26; 50 ng/mL: 1.59 ± 0.53), compared to untreated control cells. Intriguingly, the highest concentration of CCN2/CTGF treatment led to on compensatory effect and mRNA expression of *Ccn2/Ctgf* was not altered (100 ng/mL: 1.31 ± 0.65; Figure 5A). The results seen on mRNA level could also be confirmed on protein level. TGF-β1 protein synthesis was significantly increased after the treatment with 50 ng/mL and 100 ng/mL CCN2/CTGF (5 ng/mL: 1.72 ± 0.; 50 ng/mL: 1.39 ± 0.31; 100 ng/mL: 1.77 ± 0.53), compared to untreated control cells (Figure 5B). Similar results could be seen for TGF-β2, which was also significantly increased after the treatment with 50 ng/mL and 100 ng/mL CCN2/CTGF (5 ng/mL: 1.26 ± 0.96; 50 ng/mL: 1.71 ± 0.69; 100 ng/mL: 1.93 ± 0.60), compared to untreated HTM-N cells (Figure 5B). Furthermore, the auto-inductive effect of CCN2/CTGF was verified on protein level. CCN2/CTGF protein synthesis was significantly increased after the treatment with 50 ng/mL and 100 ng/mL CCN2/CTGF (5 ng/mL: 1.24 ± 0.15; 50 ng/mL: 1.47 ± 0.23; 100 ng/mL: 2.04 ± 0.94), compared to untreated control cells (1.00 ± 0.00; Figure 5B).

As we could show that CCN2/CTGF can induce TGF-β signaling *in vitro*. We analyzed the effect of CCN2/CTGF *in vivo* in our transgenic glaucoma mouse model. In anterior eye segments of 2-month-old transgenic mice, we could detect significant increases in *Tgf-b2* mRNA expression, compared to wildtype littermates (*Tgf-b2*: WT: 1.00 ± 0.16; βB1-CTGF1: 1.71 ± 0.34; Figure 5C). *Tgf-b1* mRNA expression was altered by 2 fold in comparison to the wild-type littermates, but had a higher variance in the βB1-CTGF1 mice and was not statistically significant changed (*Tgf-b1*: WT: 1.00 ± 0.15; βB1-CTGF1: 2.19 ± 0.54). Additionally, we found significantly increased protein synthesis for TGF-β2 in 2-month-old βB1-CTGF1 mice, in comparison to wildtype controls (βB1-CTGF1: 1.96 ± 0.70; Figure 5D). In immunohistochemical staining of TGF-β2 in anterior eye segment cross sections, the results obtained from real-time RT-PCR and Western blot analysis could be confirmed (Figure 5E). In wildtype animals TGF-β2 fluorescence signal was found in the iris, ciliary body, cornea, and TM. In transgenic βB1-CTGF1 mice, TGF-β2 was increased in these tissues (Figure 5E). To exclude that the increased levels of TGF-β isoforms are present in the latent form (Lawrence, 2001), and thereby would have no effect on the activity of TGF-β signaling, we analyzed the phosphorylation state of Smad2 and 3 (Figures 5F, G). The phosphorylation of both Smad2 and -3 was significantly increased in anterior eye segments of transgenic animals, compared to wildtype control mice (pSmad2: βB1-CTGF1: 1.85 ± 0.67; pSmad3: βB1-CTGF1: 1.86 ± 0.57; Figure 5F). The result could be confirmed by immunohistochemical staining against pSmad2 on anterior eye segment cross sections. In wildtype mice the pSmad2 signal was localized in the iris, ciliary body, and the TM. In βB1-CTGF1 mice the signal intensity for pSmad2 was impressively increased in the iris, ciliary body, and the TM, respectively (Figure 4G).

### 2.5 CCN2/CTGF induced TGF-β signaling activation is mediated via the Erk- and RhoA/ROCK signaling pathway

**FIGURE 6:**
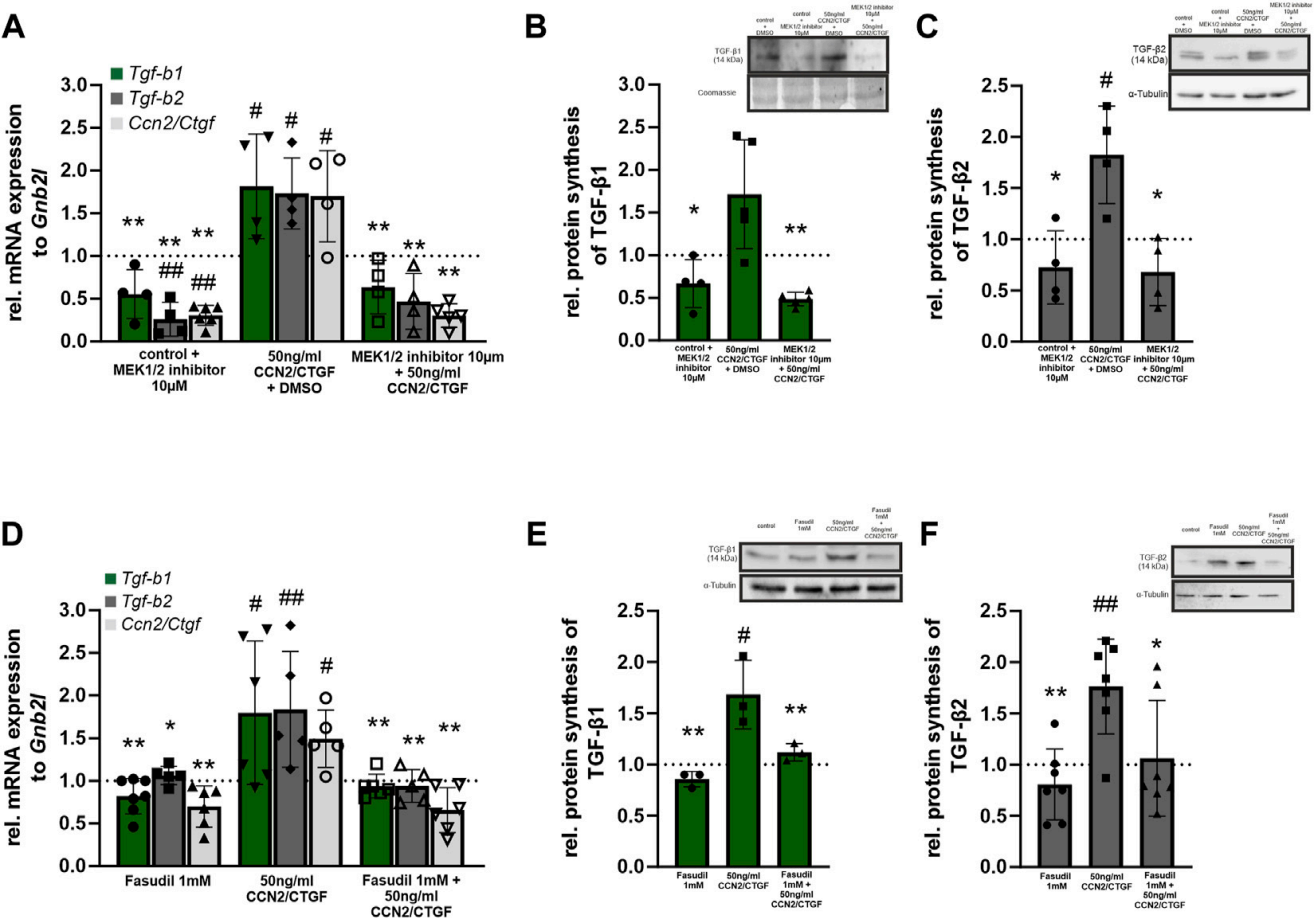
CCN2/CTGF induced TGF-β signaling activation is mediated via the Erk- and RhoA/ROCK signaling pathway. **(A)** Real-time RT-PCR analysis of *Tgf-b1*, *Tgf-b2* and *Ccn2/Ctgf* in HTM-N cells after the inhibition of the Erk-pathway. After treatment with CCN2/CTGF expression of *Tgf-b1*, *Tgf-b2*, and *Ccn2/Ctgf* was significantly higher than in DMSO treated control cells. Combined treatment with the Mek1/2 inhibitor and CCN2/CTGF did not lead to changes in expression compared to DMSO treated control cells, but the upregulation was significantly inhibited compared to cells treated with CCN2/CTGF only. *Tgf-b2* and *Ccn2/Ctgf* mRNA expression was significantly reduced after the treatment with the Mek1/2 inhibitor only, compared to DMSO treated control cells (n ≥ 4; ^#^ *p* ≤ .05, ^##^ *p* < 0.01 to DMSO control; *p ≤ 0.05, **p < 0.01 to CCN2/CTGF treatment only). **(B)** Western blot analysis of TGF-β1 in HTMN-cells after the inhibition of the Erk-pathway. After the treatment with CCN2/CTGF protein synthesis of TGF-β1 is significantly increased compared to DMSO treated control cells. Combined treatment with the Mek1/2 inhibitor and CCN2/CTGF did not lead to changes in protein synthesis compared to DMSO treated control cells, but the upregulation was significantly blocked compared to cells treated with CCN2/CTGF only (n ≥ 4, ^#^ *p* ≤ 0.05 to DMSO control; *p ≤ 0.05 to CCN2/CTGF treatment only). Right panel shows a representative Western blot. Mean value of untreated control cells was set at 1. *α*-Tubulin was used to normalize protein synthesis. **(C)** Western blot analysis of TGF-β2 in HTMN-cells after the inhibition of the Erk-pathway. After the treatment with CCN2/CTGF protein synthesis of TGF-β2 is significantly increased compared to DMSO treated control cells. Combined treatment with the Mek1/2 inhibitor and CCN2/CTGF did not lead to changes in protein synthesis compared to DMSO treated control cells, but the upregulation was significantly blocked compared to cells treated with CCN2/CTGF only (n = 4, ^#^ *p* ≤ 0.05 to DMSO control; *p ≤ 0.05 to CCN2/CTGF treatment only). Total protein stained with Coomassie was used to normalize protein synthesis. Data represented as mean ± SD. Right panel shows a representative Western blot. **(D)** Real-time RT-PCR analyses of *Tgf-b1*, *Tgf-b2*, and *Ccn2/Ctgf* in HTM-N cells after the inhibition of the RhoA/ROCK signaling pathway. After treatment with CCN2/CTGF expression of *Tgf-b1*, *Tgf-b2*, and *Ccn2/Ctgf* was significantly higher than in DMSO treated control cells. Combined treatment with Fasudil and CCN2/CTGF did not lead to changes in expression compared to DMSO treated control cells, but the upregulation was significantly inhibited compared to cells treated with CCN2/CTGF only. *Tgf-b2* and *Ccn2/Ctgf* mRNA expression was significantly reduced after the treatment with the Fasudil only, compared to DMSO treated control cells (n ≥ 5; ^#^ *p* ≤ 0.05, ^##^ *p* < 0.01 to DMSO control; *p ≤ 0.05, **p < 0.01 to CCN2/CTGF treatment only). **(E)** Western blot analysis of TGF-β1 in HTMN-cells after the inhibition of the RhoA/ROCK signaling pathway. After the treatment with CCN2/CTGF protein synthesis of TGF-β1 is significantly increased compared to DMSO treated control cells. Combined treatment with Fasudil and CCN2/CTGF did not lead to changes in protein synthesis compared to DMSO treated control cells, but the upregulation was significantly blocked compared to cells treated with CCN2/CTGF only (n = 3, ^#^ *p* ≤ 0.05 to DMSO control; **p < 0.01 to CCN2/CTGF treatment only). Right panel shows a representative Western blot. Mean value of untreated control cells was set to1. *α*-Tubulin was used to normalize protein synthesis. **(F)** Western blot analysis of TGF-β2 in HTMN-cells after the inhibition of the RhoA/ROCK signaling pathway. After the treatment with CCN2/CTGF protein synthesis of TGF-β2 was significantly increased compared to DMSO treated control cells. Combined treatment with Fasudil and CCN2/CTGF did not lead to changes in protein synthesis compared to DMSO treated control cells, but the upregulation was significantly blocked compared to cells treated with CCN2/CTGF only (n = 7, ^##^ *p* < 0.01 to DMSO control; *p ≤ 0.05, **p < 0.01 to CCN2/CTGF treatment only). Total protein stained with Coomassie was used to normalize protein synthesis. Data represented as mean ± SD. Right panel shows a representative Western blot. Dotted line indicates the control group treated with the solvent of the inhibitor for the appropriate experiment. For statistical analysis a one-way ANOVA was performed.

In a previous publication we could show that CCN2/CTGF activates the RhoA/ROCK and Erk signaling pathway in human TM cells (Junglas et al., 2012). To investigate which pathways are responsible for the CCN2/CTGF induced increase in TGF-β signaling, we performed cell culture experiments with HTM-N cells in which we blocked the signaling pathway of the Erk1/2 kinase via the specific inhibition of the Mek1/2 kinase and inhibition of the Rho-associated protein kinase (ROCK) with Fasudil. To analyze if the Erk-pathway is involved in the CCN2/CTGF induced activation of TGF-β signaling, we performed real time RT-PCR analysis for *Tgf-b1* and *Tgf-b2*, as well as *Ccn2/Ctgf* mRNA expression. Therefore, HTM-N cells were either treated with the Mek1/2 inhibitor, 50 ng/mL CCN2/CTGF, or with the combination of CCN2/CTGF and the Mek1/2 inhibitor. Control cells were treated with DMSO. *Tgf-b1* mRNA expression was significantly decreased after the treatment with the Mek1/2 inhibitor, compared to DMSO treated control cells (0.56 ± 0.28). CCN2/CTGF treatment alone significantly increased *Tgf-b1* mRNA expression, as we could already show in Figure 4 (1.82 ± 0.61). The inhibition of Erk signaling in combination with CCN2/CTGF treatment diminished *Tgf-b1* mRNA expression, and therefor blocked the CCN2/CTGF induced effect on *Tgf-b1* expression (0.64 ± 0.32; Figure 6A). Similar results could be seen for *Tgf-b2* expression. The treatment with Mek1/2 inhibitor alone led to a significant decrease of *Tgf-b2* mRNA expression, compared to DMSO treated control cells (0.26 ± 0.20). After CCN2/CTGF treatment alone *Tgf-b2* mRNA expression was significantly increased (1.73 ± 0.42). In combination with the Mek1/2 inhibitor, the CCN2/CTGF inducible effect was blocked, and *Tgf-b2* mRNA expression was significantly reduced (0.47 ± 0.33, Figure 6A). Interestingly, the auto-induction of CCN2/CTGF was also blocked by inhibiting the Erk signaling pathway, as the combination of Mek1/2 inhibitor and CCN2/CTGF treatment significantly decreased *Ccn2/Ctgf* mRNA levels (0.3 ± 0.11). Furthermore, Mek1/2 inhibitor alone could reduce *Ccn2/Ctgf* mRNA expression (0.31 ± 0.08), whereas CCN2/CTGF treatment increased *Ccn2/Ctgf* mRNA expression (1.7 ± 0.55; Figure 6A). As this study focuses on the effect of CCN2/CTGF on TGF-β signaling, we performed Western blot analysis only for TGF-β1 and TGF-β2. This analysis could confirm the results we obtained for mRNA expression. TGF-β1 protein synthesis was significantly reduced after the treatment with Mek1/2 inhibitor in combination with CCN2/CTGF compared to CCN2/CTGF treatment (0.49 ± 0.08). Furthermore, Mek1/2 inhibitor alone could reduce TGF-β1 level (0.67 ± 0.28), whereas CCN2/CTGF treatment increased TGF-β1 synthesis (1.72 ± 0.64; Figure 6B). Similar results could be observed for TGF-β2. The TGF-β2 levels were significantly decreased after the combined treatment with CCN2/CTGF and the Mek1/2 inhibitor, compared to CCN2/CTGF treated cells (0.68 ± 0.33). In contrast, the treatment with the inhibitor alone did not alter TGF-β2 protein synthesis (0.72 ± 0.36), whereas the inductive effect of CCN2/CTGF could again be proved by the single treatment (1.82 ± 0.47; Figure 6C). To investigate if the RhoA/ROCK signaling pathway is involved in CCN2/CTGF mediated induction of TGF-β1, TGF-β2 and CCN2/CTGF, we inhibited the signaling pathway by blocking the ROCK kinase with its specific inhibitor Fasudil. Therefore, HTM-N cells were either treated with the Fasudil, 50 ng/mL CCN2/CTGF, or with the combination of CCN2/CTGF and Fasudil. Untreated cells served as a control. We performed real time RT-PCR analysis for *Tgf-b1* and *Tgf-b2*, as well as *Ccn2/Ctgf*. *Tgf-b1* mRNA expression was significantly reduced after the treatment with a combination of CCN2/CTGF and Fasudil (Fasudil: 0.82 ± 0.21; 50 ng/mL CCN2/CTGF: 1.80 ± 0.84; 50 ng/CCN2/CTGF + Fasudil: 0.94 ± 0.14), compared to CCN2/CTGF treated cells (Figure 6D). Similar results were obtained for *Tgf-b2.* mRNA expression of *Tgf-b2* was significantly reduced after the treatment with a combination of CCN2/CTGF and Fasudil (Fasudil: 1.06 ± 0.10; 50 ng/mL CCN2/CTGF: 1.84 ± 0.68; 50 ng/CCN2/CTGF + Fasudil: 0.94 ± 0.19), compared to CCN2/CTGF treated cells (Figure 6D). Interestingly, the auto-inductive effect of CCN2/CTGF could also be blocked by the inhibition of the RhoA/ROCK pathway. *Ccn2/Ctgf* mRNA levels were significantly reduced after the treatment with a combination of CCN2/CTGF and Fasudil (Fasudil: 0.74 ± 0.35; 50 ng/mL CCN2/CTGF: 1.46 ± 0.26; 50 ng/CCN2/CTGF + Fasudil: 0.73 ± 0.42), compared to CCN2/CTGF treated cells (Figure 6D). Finally, we performed Western blot analysis for TGF-β1 and TGF-β2, which could verify the results obtained from real time RT-PCR analysis. TGF-β1 protein synthesis was significantly reduced after the treatment with a combination of CCN2/CTGF and Fasudil (Fasudil: 0.86 ± 0.08; 50 ng/mL CCN2/CTGF: 1.68 ± 0.33; 50 ng/CCN2/CTGF + Fasudil: 1.12 ± 0.09), compared to CCN2/CTGF treated cells (Figure 6E). Protein levels of TGF-β2 were significantly reduced after the treatment with a combination of CCN2/CTGF and Fasudil (Fasudil: 0.81 ± 0.35; 50 ng/mL CCN2/CTGF: 1.76 ± 0.46; 50 ng/CCN2/CTGF + Fasudil: 1.06 ± 0.57), compared to CCN2/CTGF treated cells (Figure 6F).

## 3 Discussion

We conclude that CCN2/CTGF is an important factor in the homeostatic balance of growth factors in the anterior eye segment. The overexpression of CCN2/CTGF inhibits the BMP pathway and tips the balance towards TGF-β2, thereby contributing either to developmental malformations or to the pathological processes in POAG. The conclusion rests on our observation that 1) high CCN2/CTGF expression leads to developmental malformations of the ciliary body due to blocking of the BMP signaling pathway, 2) CCN2/CTGF directly attenuates BMP signaling in the TM *in vitro* and *in vivo*, and 3) CCN2/CTGF increases TGF-β signaling in the TM *in vitro* and *in vivo*.

The tight control and the fragile balance of the growth factors could be shown by our two transgenic mouse models. While the moderately expressing βB1-CTGF1 line showed no alterations during development, a high expression of CCN2/CTGF could disrupt ciliary body morphogenesis by inhibiting the BMP signaling in the ciliary body. The important role of BMPs and their signaling pathway during eye development was proven by many publications (Dudley et al., 1995; Luo et al., 1995; Jena et al., 1997; Furuta and Hogan, 1998; Wawersik et al., 1999; Zhao et al., 2002), especially for *Bmp-4* and *Bmp-7.* Both BMPs are involved in lens and ciliary body development (Zhao et al., 2002). In βB1-CTGF6 mice we observed morphological defects in the ciliary body at P1, leading to failure in ciliary epithelium folding during ciliary processes formation. The same effect was described in lens specific Noggin-overexpressing mice, also showing morphological malformation in the folding of the ciliary body. The developmental abnormalities were due to a reduced BMP signaling in the epithelial layers of ciliary body (Zhao et al., 2002) similar to what we could observe in the βB1-CTGF6 mice. One could speculate that the BMP inhibiting effect of CCN2/CTGF should also affect lens development as BMPs are required for a proper lens formation, but the used βB1-crystallin promoter has its strong activity starting at embryonic day 12.5. At this timepoint there is already a decline of BMP-4 and -7 expression in the developing lens (Huang et al., 2015).

The *in vitro* experiments could show that the CCN2/CTGF effect is a primary effect and not a secondary inhibition occurring during the developmental process. The inhibitory potential against the BMP signaling pathway by CCN2/CTGF was comparable to the effect of Noggin, a specific BMP inhibitor, in human TM cells. The interaction with BMPs was described at the vWC domain of CCN2/CTGF, preventing the binding of BMPs to the BMP receptors (Abreu et al., 2002). This potential interaction could be the cause of the marked reduction of the BMP signaling in the ciliary body of βB1-CTGF6 mice. It is of interest that during ocular development a high and persistent CCN2/CTGF promotor activity in neural crest cells was described (Dillinger et al., 2021), leading to the suggestion that CCN2/CTGF might play an important role in the specification and determination of ciliary body fate by regulating BMP activity, but therefore extensive developmental studies have to be performed.

**FIGURE 7:**
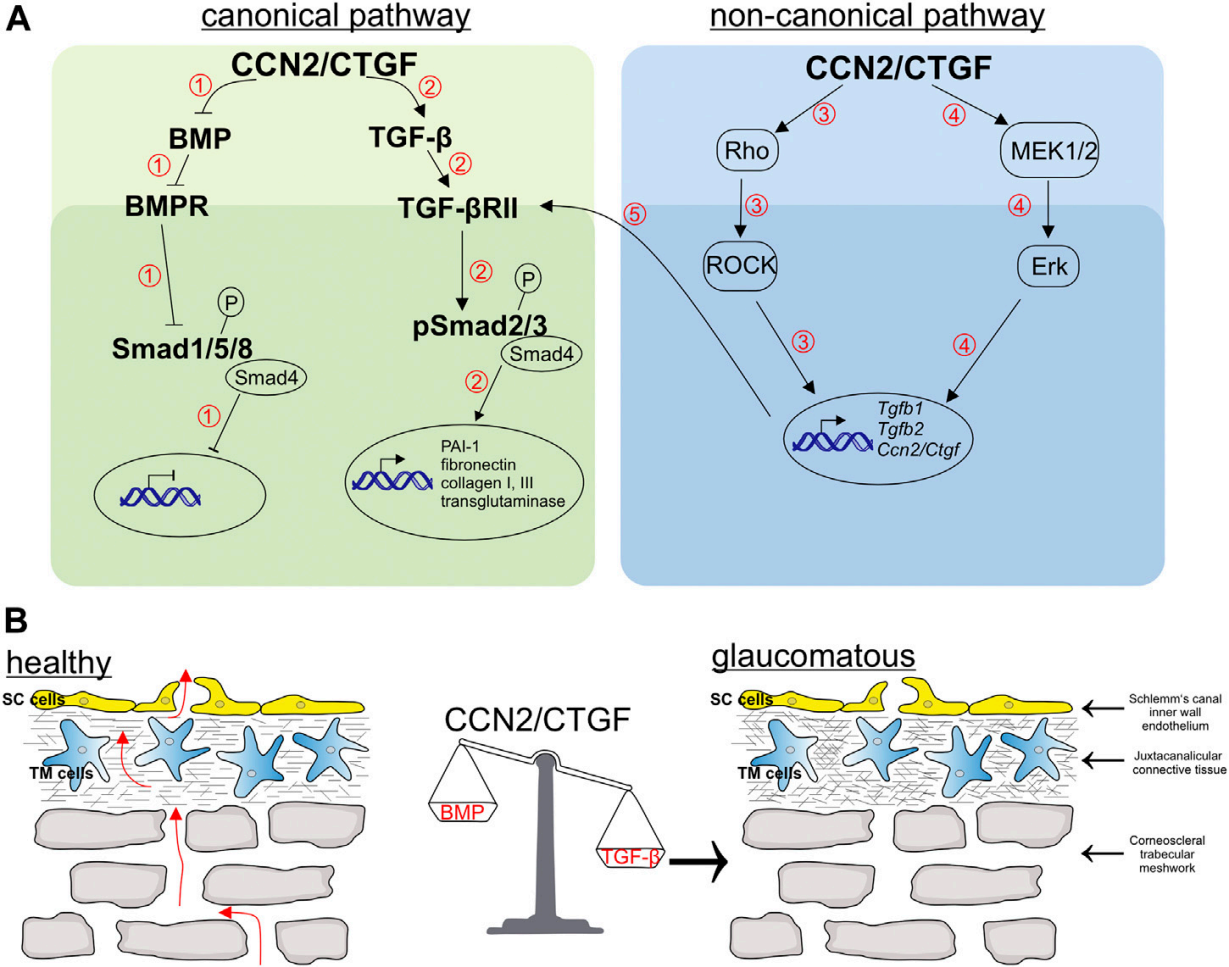
Schematic illustration of pathways regulated by CCN2/CTGF and related changes in the aqueous humor outflow pathway. **(A)** CCN2/CTGF regulates the BMP (1) and TGF-β (2) signaling via the Smad-dependent pathway. 1) The overexpression of CCN2/CTGF leads to the inhibition of BMP ligands and the blocking of the phosphorylation of the downstream signaling molecule Smad1/5/9, which thereby inhibits gene transcription of BMP target genes. 2) In contrast, CCN2/CTGF can induce ligands of TGF-β signaling like TGF-β1 and TGF-β2. This leads to enhanced levels of phosphorylated Smad2 and Smad3, which finally enable the gene transcription of target genes, like PAI-1, fibronectin, Collagen type I and III, and transglutaminase. Furthermore, CCN2/CTGF induces TGF-β1 and TGF-β2 via Smad-independent pathways, the Rho/ROCK 3) and Erk-pathway 4). This increased levels of TGF-βs can additionally act via the Smad-dependent pathway to increase target genes 5). **(B)** Schematic drawing of the trabecular meshwork, which contains the uveal trabecular meshwork (not shown), the corneoscleral trabecular meshwork and the juxtacanalicular trabecular meshwork. The aqueous humor passes the different layers of the trabecular meshwork und finally passes the pores, build up in the inner wall of Schlemm’s canal by Schlemm’s canal endothelial cells. CCN2/CTGF can tip the balance of growth factors towards TGF-β, by its action in the related pathways explained in **(A)**. The imbalance in growth factors leads to an increase in extracellular matrix in the trabecular meshwork and an enhanced contractility of trabecular meshwork cells. These alterations finally lead to a decreased outflow facility and an increased intraocular pressure as seen in primary open angle glaucoma.

In the CCN2/CTGF overexpressing mouse model with the moderate expression (βB1-CTGF1) no morphological alterations during ocular development were observed (Junglas et al., 2012). Interestingly, the moderate expression of CCN2/CTGF is sufficient to modulate the TGF-β and BMP signaling pathway, causing a POAG like pathogenesis in the transgenic animals (Figure 7). The mouse models show that there are dose dependent effects of CCN2/CTGF within the anterior eye segment, which acts as a modulator of the homeostatic balance of the TGF-β and BMP signaling pathway, which is disrupted in POAG. The TGF-β and BMP pathways share similarities in their signaling patterns. Ligands of the BMP signaling pathway bind to their receptors, which leads to the phosphorylation of Smad1, 5 and 8 (pSmad). pSmad1/5/8 form a complex with Smad4, translocate into the nucleus and activate transcription of target genes. TGF-β leads by binding to its specific TGF-β receptors to a phosphorylation of Smad2 and 3, forming a complex with Smad4 to translocate into the nucleus and activate gene transcription. The regulatory mechanism between both growth factors is complex and was the topic of multiple studies in the TM (Abreu et al., 2002; Wordinger et al., 2002; Fuchshofer et al., 2007; Wordinger et al., 2007; Fuchshofer et al., 2009). It was shown that at the receptor level BAMBI, a pseudo-receptor of the TGF-β superfamily, is involved in the dysregulation contributing to the ECM changes in the TM (Hernandez et al., 2018), whereas in the canonical signaling pathway the inhibitory Smad7 is able to regulate both pathways in the TM cells. Following Smad7 inhibition the antagonizing effect of BMP-7 on TGF-β induced CCN2/CTGF expression was abolished (Fuchshofer et al., 2009). On the transcriptional site the induction of *Id1* and *Id3*, target genes of the BMP signaling pathway, could prevent a TGF-β induced IOP elevation (Mody et al., 2021).

In the aqueous humor of patients with POAG, one of the best-described molecular change is an increased concentration of TGF-β2, whereas TGF-β1 was not altered (Ochiai and Ochiai, 2002), in contrast to patients with pseudoexfoliation glaucoma, who have increased concentration of TGF-β1 and no changes in TGF-β2 (Schlötzer-Schrehardt et al., 2001). Many follow-up studies have demonstrated that pathological changes in the outflow pathways can be triggered by TGF-β2. The pathological changes of the ECM in the TM of POAG patients, as well as the increased contractility and stress fiber formation, are probably influenced by an increased concentration of TGF-β2 (Rodrigues et al., 1980; Babizhayev and Brodskaya, 1989; Tamm et al., 1996; Nakamura et al., 2002).Many of the TGF-β induced changes are mediated by CCN2/CTGF. CCN2/CTGF itself is present in the aqueous humor and has been described to be increased in patients with POAG and pseudoexfoliation glaucoma (van Setten et al., 2002; Ho et al., 2005; Browne et al., 2011; Ho et al., 2020). Glaucomatous Schlemm`s canal cells had a significantly higher CCN2/CTGF expression than control cells derived from healthy donors (Overby et al., 2014). Mice with an increase in TGF-β or CCN2/CTGF by viral gene transfer or by transgenic overexpression show a reduction in outflow facility and thereby develop an elevated IOP (Shepard et al., 2010; Junglas et al., 2012; McDowell et al., 2013). Interestingly, we could observe enhanced levels of TGF-β1 and TGF-β2 in the transgenic βB1-CTGF1 mice accompanied by increased TGF-β signaling in the outflow tissues, which would make CCN2/CTGF of interest for pathological effects in both pseudoexfoliation glaucoma and POAG patients. We could prove that the increase of TGF-βs and their signaling pathway is a direct CCN2/CTGF effect in immortalized human TM cells. Besides, we could show that CCN2/CTGF is able to induce itself in immortalized human TM cells *in vitro*. It can be suggested that the distinct immunoreactivity of CCN2/CTGF, present in the trabecular meshwork in βB1-CTGF1 mice, in contrast to wildtype littermates, is caused by a similar auto-inductive effect of the increased amounts in the aqueous humor (Junglas et al., 2012). The stimulating effect of CCN2/CTGF and TGF-β2 on ECM synthesis is markedly antagonized by BMP-4 and -7 (Fuchshofer et al., 2007; Wordinger et al., 2007). In the murine anterior eye segment, we observed the BMP-7 signal predominantly in the ciliary body and the BMP-4 signal mainly in the TM and that a moderate CCN2/CTGF overexpression is sufficient to reduce the BMPs in these tissues. Furthermore, we found an increase of the BMP inhibitor Gremlin in the outflow tissue of our transgenic βB1-CTGF1 mouse model. Gremlin is increased in the aqueous humor of POAG patients (Wordinger et al., 2002) and increased concentrations of Gremlin led to an increased outflow resistance in *ex vivo* ocular organ culture (Wordinger et al., 2007) and in mice (McDowell et al., 2015).

There are numerous molecular pathways discussed to be involved in the pathogenesis of POAG (Wang et al., 2018). Our results showed that CCN2/CTGF leads to a downregulation of BMPs and an upregulation of TGF-βs, which in turn caused a modulation of the canonical pathways of these growth factors. However, whether the CCN2/CTGF effect would also lead to changes in the non-canonical pathways and whether these pathways are regulated independently or intertwine with each other must be elucidated in further studies (Figure 7). Our previous publication showed that CCN2/CTGF utilizes the ERK and RhoA/ROCK signaling pathway in the TM and in the βB1-CTGF1 the treatment with fasudil, a ROCK inhibitor, caused a reduction in IOP (Junglas et al., 2012). The RhoA/ROCK pathway was identified to be a promising target for the development of new drugs in POAG. The activation of the RhoA/ROCK pathway causes marked changes in the stress fiber formation, contractility and stiffness in the outflow pathway leading to an increase in outflow resistance. ROCK inhibitors are the first drugs, which directly act on the outflow tissues reducing the outflow resistance and the IOP in POAG patients (Tanna and Johnson, 2018). Our analysis showed that CCN2/CTGF mediates its inductive effect on TGF-β signaling via these signaling pathways. It is known that the activation of the ERK and the RhoA/ROCK signaling pathway leads to increased levels of fibronectin, *α*-smooth muscle actin and the enhanced formation of actin stress fibers in human TM cells (Pattabiraman and Rao, 2010) and the observed increase of TGF-β could augment these effects, causing a vicious circle of amplifying mechanisms in the outflow tissues.

Finally, we discovered a complex network of molecular factors in which CCN2/CTGF is embedded. All the investigated factors are present in the anterior eye segment in the healthy eye, and we do not know what might cause the disruption of the homeostatic balance of these growth factors. As POAG is an age dependent disease, it could be speculated that with increasing age higher amounts of TGF-β2 might also have beneficial effects in ocular tissues and thereby causing the side effects of increased ECM production, higher contractility and increased stiffness, leading on the long run to an increased IOP. We conclude, that CCN2/CTGF functions as a modulator of the homeostatic balance of BMP and TGF-β signaling pathways. In POAG an overexpression of CCN2/CTGF tips this balance towards TGF-β accompanied with an attenuation of BMP signaling in the trabecular meshwork, whether this can be prevented by new therapeutic drugs like the Rho kinase inhibitors will be the topic of future studies.

## 4 Materials and methods

### 4.1 Animals

Transgenic βB1-CTGF1 and βB1-CTGF6 mice were generated as described previously (Junglas et al., 2012) and compared to wildtype littermates. In brief, in these mouse lines, the chicken βB1-crystallin promotor was used to direct high and specific expression of transgenes to lens fibers of the mouse eye (Duncan et al., 1996; Junglas et al., 2012). As the βB1-crystallin promoter is lens specific, the overexpression of CCN2/CTGF was only detected in the lenses of the βB1-CTGF lines not in other ocular tissues (Junglas et al., 2012). The experiments in this study was performed with mice of both sex and in the analyses the sex was not taken into account. In total 56 mice were used for the experiments (immunohistochemical studies = 15, molecular biology = 35, SEM = 6). Mice were housed under standardized conditions of 63% air humidity and 21°C room temperature. Feeding was *ad libitum*. Animals were kept at a 12 h light/dark cycle (6 a.m.–6 p.m.). All procedures conformed to the tenets of the National Institutes of Health Guidelines on the Care and Use of Animals in Research, the EU Directive 2010/63/E and the ARVO Statement for the Use of Animals in Ophthalmic and Vision Research and were approved by the local authorities (54-2532.1-44/12; Regierung Oberpfalz, Bavaria, Germany).

### 4.2 Cell culture methods

Human immortalized trabecular meshwork (HTM-N) (Pang et al., 1994) cells were cultured in DMEM with 10% FCS, penicillin (100 U/mL) and streptomycin (100 μg/mL) on 6-well plates. Different treatments in this study were performed as follows: 1) To investigate the BMP signaling activity in HTM-N cells, cells were treated either with 10 ng/mL BMP-4 (solubilized in 1x PBS) or 10 ng/mL BMP-7 (solubilized in 1x PBS) for 1, 3, 6 or 24 h. Control cells were treated with 1x PBS. 2) To analyze the effect of CCN2/CTGF on TGF-β signaling activity, HTM-N cells were treated with 5 ng/mL, 25 ng/mL, 50 ng/mL or 100 ng/mL CCN2/CTGF (solubilized in 1x PBS) for 24 h.Control cells were treated with 1x PBS. 3) To assess if CCN2/CTGF regulates the expression of TGF-β1, TGF-β2 and CCN2/CTGF via the Erk-pathway in HTM-N cells, cells were treated with 50 ng/mL CCN2/CTGF only or in combination with the Mek1/2 inhibitor (UO126; Bioline, Luckenwalde, Germany). Cells treated with DMSO (Roth, Karlsruhe, Germany) served as control, since the Mek1/2 inhibitor was solubilized in DMSO. 4) To investigate if CCN2/CTGF regulates the expression of TGF-β1, TGF-β2 and CCN2/CTGF via the RhoA/ROCK pathway in HTM-N cells, cells were treated with CCN2/CTGF only or in combination with the RhoA/ROCK signaling pathway inhibitor, Fasudil (solubilized in 1x PBS) (HA-1077; Sigma-Aldrich, Taufkirchen, Germany). Control cells were treated with 1x PBS. Each experiment was individually performed at least three times or more.

### 4.3 RNA analysis

Total RNA was extracted either from HTM-N cells or anterior eye segments of 2 month old transgenic βB1-CTGF1and wildtype mice, including the cornea, iris, ciliary body and TM, with TriFast™(Peqlab, Erlangen, Germany) according to the manufacturer`s recommendations. In brief, 500 µL of TriFast™ was added to the tissue of the anterior eye segment and homogenized by a ULTRA-TURRAX (IKA®- GmbH, Staufen, Germany) and for the isolation of cell culture samples 500 µL TriFast™ was added to each well and cells were removed by using a cell scraper. The following steps were performed the same way for mouse tissue samples and cell culture samples. First, 100 µL chloroform was added, vortexed for 15 s and centrifuged for 20 min at 12,000 g at 4°C to induce phase separation. The aqueous phase containing the total RNA was transferred into a new tube, precipitated with 100% isopropanol, washed twice with 75% ethanol and dissolved in RNase-free water. Real-time RT-PCR was performed from total RNA using the qScriptTM cDNA Synthesis Kit (Quanta Biosciences, Gaithersburg, United States) according to the manufacturer´s introductions. Real-time RT-PCR was performed on a BioRad iQ5 Real-time PCR Detection iCycler System (BioRad, Munich, Germany) with the temperature profile as follows: 50 cycles of 20 s melting at 94°C, 10 s of annealing at 60°C and 20 s of extension at 70°C. All primers were purchased from Invitrogen and extended over exon-intron boundaries: (*mmBmp4* forward) 5′-gat​ctt​tac​cgg​ctc​cag​tct-3’; (*mmBmp4* reverse) 5′-tgg​gat​gtt​ctc​cag​atg​ttc-3’; (*mmBmp7* forward) 5′-cga​gac​ctt​cca​gat​cac​agt-3’; (*mmBmp7* reverse) 5′-cac​caa​gaa​gag​ctc​cga​ct-3’; (*huCcn2/Ctgf* forward) 5′-ctc​ctg​cag​gct​aga​gaa​gc-3’; (*huCcn2/Ctgf* reverse) 5′-gat​gca​ctt​ttt​gcc​ctt​ctt-3’; (*huGnb2l* forward) 5′-gct​act​acc​ccg​cag​ttc​c-3’; (*huGnb2l* reverse) 5′-cag​ttt​cca​cat​gat​gat​ggt​c-3’; (*mmGnb2l* forward) 5′-tct​gca​agt​aca​cgg​tcc​ag-3’; (*mmGnb2l* reverse) 5′-gag​acg​atg​ata​ggg​ttg​ctg-3’; (*mmGremlin* forward) 5′-gac​cca​cgg​aag​tga​cag​a-3’; (*mmGremlin* reverse) 5′-ccc​tca​gct​gtt​ggc​agt​ag-3’; (*huTgfβ-1* forward) 5′-gca​gca​cgt​gga​gct​gta-3’; (*huTgfβ-1* reverse) 5′-cag​ccg​gtt​gct​gag​gta-3’; (*mmTgfβ-1* forward) 5′-tgg​agc​aac​atg​tgg​aac​tc-3’; (*mmTgfβ-1* reverse) 5′-gtc​agc​agc​cgg​tta​cca-3’; (*huTgfβ-2* forward) 5′-cca​aag​ggt​aca​atg​cca​ac-3’; (*huTgfβ-2* reverse) 5′-cag​atg​ctt​ctg​gat​tta​tgg​tat​t-3’; (*mmTgfβ-2* forward) 5′-tct​tcc​gct​tgc​aaa​acc-3’; (*mmTgfβ-2* reverse) 5′-gtg​gga​gat​gtt​aag​tct​ttg​ga-3’; (*mmSmad2* forward) 5′-agg​acg​gtt​aga​tga​gct​tga​g-3’; (*mmSmad2* reverse) 5′-gtc​ccc​aaa​ttt​cag​agc​aa-3’; (*mmSmad3* forward) 5′-tca​aga​aga​cgg​ggc​agt​t-3’; (*mmSmad3* reverse) 5′-ccg​acc​atc​cag​tga​cct-3’; (*mmSmad6* forward) 5′-gtt​gca​acc​cct​acc​act​tc-3’; (*mmSmad6* reverse) 5′-gga​gga​gac​agc​cga​gaa​ta-3’; (*mmSmad7* forward) 5′-ccc​aat​gga​ttt​tct​caa​acc-3’; (*mmSmad7* reverse) 5′-ggg​cca​gat​aat​tcg​ttc​c-3’. RNA that was not reverse transcribed served as negative control. For relative quantification of the experiments, *Gnb2l* and *Rpl32* were used as housekeeping genes. BioRad iQ5 Optical System Software (version 2.0) was used for analysis and ΔΔct-method was applied for normalization.

### 4.4 Western blot analysis

Proteins were isolated following the RNA isolation according to the manufacturer`s instructions with TriFast™ (Peqlab, Erlangen, Germany). After removing the aqueous phase, containing the RNA, the remaining organic phase was used to isolate proteins. In brief, the proteins were precipitated with isopropanol, washed three times with Guanidine hydrochloride and were washed once with 95% ethanol. Finally, the proteins were dissolved in 1% SDS containing protease (Serva Electrophoresis GmbH, Heidelberg, Germany) and phosphatase inhibitor (Sigma-Aldrich, Taufkirchen, Germany). Protein concentration was determined by the bicinchoninic acid assay (Interchim, Montlucon, Cedex, France). Proteins were separated by SDS-PAGE and transferred to polyvinylidene difluoride (PVDF) membranes (Roche, Mannheim, Germany). Western blot analysis was performed with specific antibodies as described previously (Fuchshofer et al., 2005). Specific antibodies were used as follows: rabbit anti-pSmad1/5/8 (1:1,000, Cell Signaling Technology, Danvers, MA, United States; RRID:AB_331671), goat anti-BMP7 (1:500, Santa Cruz Biotechnology; RRID:AB_2227926), goat anti-BMP4 (1:500, Santa Cruz Biotechnology; RRID:AB_2243391), rabbit anti-Gremlin (1:200, Santa Cruz Biotechnology; RRID:AB_2279266), rabbit anti-TGF- β1 (1:200, Promega), rabbit anti- TGF- β2 (1:200; Santa Cruz Biotechnology), rabbit anti-α-Tubulin (1:2500, Rockland; RRID:AB_2612816), rabbit anti-pSmad2 (1:200, Cell Signaling Technology; RRID:AB_390732), rabbit anti-pSmad3 (1:200, Cell Signaling Technology; RRID:AB_2193207), rabbit anti-GAPDH-HRP (1:5,000, Cell Signaling Technology; RRID:AB_1642205), rabbit anti-LaminB1 (1:1,000; Cell signaling Technology; Cat# 13435, RRID:AB_2737428), chicken anti-goat (HRP, 1:2000, Santa Cruz Biotechnology), chicken anti-goat (AP, 1:2000, Santa Cruz Biotechnology), chicken anti-rabbit (HRP, 1:2000 Cell Signaling Technology) and chicken anti-rabbit (AP, 1:2000, Santa Cruz Biotechnology). *α*-Tubulin, GAPDH and total protein staining with Coomassie were used as loading control to normalize the signal intensity of the Western blots. LaminB1 was used as loading control for Western blot application with nuclear fraction to normalize the signal intensity. The intensity of the bands detected by Western blot analysis was determined using appropriate software (AIDA Image analyzer software, Raytest).

### 4.5 Isolation of cytoplasmic and nuclear fraction

For the analysis of pSmad1/5/8 in BMP-4 and BMP-7 treated HTM-N cells the cytoplasmic and nuclear protein fraction was isolated separately. To do so the NE-PER Nuclear and Cytoplasmic Extraction Reagent from Thermo Fisher was used and the proteins were isolated to the manual instructions. In brief, treated HTM-N cells were harvest with trypsin-EDTA, centrifuged and washed with x PBS. Ice-cold CER I was added to the cell pellet, vortexed and incubated on ice for 10 min. Next, CER II was added to the tube and the samples were vortexed and incubated on ice again. Cell fractions were separated by the next centrifugation step. Supernatant (cytoplasmic fraction) was immediately transferred to a new tube and stored at −80°C. The insoluble pellet, containing the nuclei was suspend in ice-cold NER. For the next 40 min the samples were incubated on ice and vortexed for 15 s every 10 min. After the final centrifugation step the supernatant now containing the nuclear fraction was transferred to a new tube and stored at −80°C.

### 4.6 Immunocytochemistry

HTM-N cells were seeded on coverslips and treated with either with 10 ng/mL BMP-4, 10 ng/mL BMP-7 or left untreated as controls for 24 h under serum free conditions. Cells were washed twice with PBS, fixed with 4% (w/v) paraformaldehyde (PFA) for 5 min and washed again three times with PBS. After blocking with 1% bovine serum albumin (BSA), 0.2% cold water fish gelatin (CWFG, Sigma-Aldrich), 0.1% Triton-X in 1x TBS, cells were stained with rabbit anti-pSmad1/5/8 (1:100, Cell Signaling Technology, Danvers, MA, United States; RRID: AB_331671) at 4°C overnight. After washing three times with 1x TBS, slides were incubated in Alexa Fluor^®^488 (1:1,000) for 1 h at RT. As a control for unspecific binding of the secondary antibody, negative controls were performed. Finally, 4,6-diamidino-2-phenylindole (DAPI) (Vector Laboratories, Burlingame, CA, United States) was added to counterstain nuclear DNA. Slides were dried overnight and visualized using a Zeiss Axio Imager fluorescence microscope (Carl Zeiss AG, Jena, Germany).

### 4.7 Immunohistochemistry

Eyes of 2 month old βB1-CTGF1 transgenic and wildtype mice were enucleated and fixed in 4% (w/v) PFA for 24 h. The eyes were equilibrated in 10%, 20% and 30% sucrose, embedded in Tissue Tek optimal cooling temperature compound (Sakura Finetek Europe B.V., Zoeterwounde, Netherland), and stored at −20°C. Frozen 12 µm cross sections were cut on the cryostat. After blocking with 1% bovine serum albumin (BSA), 0.2% CWFG(Sigma-Aldrich), 0.1% Triton-X in 0.1 M phosphate buffer or 1x TBS (for pSmad1/5/8 and pSmad2) for 1 h at RT. Afterwards sections were incubated with the primary antibody as follows: goat anti-BMP-4 (1:50, Santa Cruz Biotechnology; RRID:AB_2243391), goat anti- BMP-7 (1:50, Santa Cruz Biotechnology; RRID:AB_2227926), rabbit anti-Gremlin (1:50, Santa Cruz Biotechnology; RRID:AB_2279266), rabbit anti-pSmad1/5/8 (1:100, Cell Signaling Technology, Danvers, MA, United States; RRID:AB_331671), rabbit anti-pSmad2 (1:50, Cell Signaling Technology; RRID:AB_390732) and rabbit anti-TGF-β2 (1:50, Cell Signaling Technology) at 4°C overnight. As a next step, sections were washed three times with 0.1 M phosphate buffer or 1x TBS (for pSmad1/5/8 and pSmad2), followed by the incubation with the secondary antibody as follows: Cy™3 donkey anti-goat (1:2000), Alexa Fluor^®^488 goat anti-rabbit (1:1,000) and Cy™3 goat anti-rabbit (1:2,000) for 1 h at RT. As a control for unspecific binding of secondary antibodies, negative controls were performed. After washing three times with 0.1 M phosphate buffer or1x TBS (for pSmad1/5/8 and pSmad2), the slides were mounted with DAPI 1:10 (Dako). Slides were dried overnight and visualized using a Zeiss Axio Imager fluorescence microscope (Carl Zeiss AG, Jena, Germany).

### 4.8 Wholemount preparation of the ciliary body

Eyes of 1 month old were fixed in 4% PFA for 3 h at 4°C. The bulbus was cut open along the ora serrata. The lens and remnants of the retina were removed. The cornea was cut open and the iris was carefully removed without harming the ciliary body, modified after Thomson and Quaggin (2018). The tissue was additionally fixed in 100% methanol for 20 min at −20°C. Wholemounts were pretreated with 50 mM NH_4_Cl for 1 h and 0.5% Triton X-100 for 30 min at RT. As blocking solution, we used 2% BSA, 0.2% CWFG, 0.1% Triton X-100 in 0.1 M phosphate buffer, which was applied for 1 h at RT. The first antibody goat anti-cluster of differentiation 31 (CD31) (1:100, R&D Systems; Cat# AF3628, RRID: AB_2161028) was incubated at 4°C over night. Afterwards, wholemounts were washed 3 times for 10 min with 0.1 M phosphate buffer and then incubated with donkey anti-goat Alexa 647 (1:1,000, Thermo Fisher Scientific Cat# A-21447, RRID: AB_2535864) for 2 h at RT. After washing 2 times with 0.1 M phosphate buffer and once with H2O a.d. for 10 min each, wholemounts were mounted on an object slide with mowiol (Carl Roth, Germany).

### 4.9 Light microscopy

For light electron microscopy, eyes were obtained from βB1-CTGF6 and wildtype mice on postnatal day (P) 1, 6, 12, 18 and 21. Eyes were enucleated and fixed with Karnovsky’s solution (2.5% glutaraldehyde and 2.5% paraformaldehyde in 0.1 M cacodylate buffer) for 24 h (Karnovsky, 1965). After rising in 0.1 M cacodylate buffer, postfixation was accomplished in a mixture of 1% OsO4 and 0.8% potassium ferrocyanide in 0.1 M cacodylate buffer for 2 h at 48°C. After dehydration in a graded series of ethanol, the eyes were embedded in Epon (Serva, Heidelberg, Germany). Semithin sections (1 µm) were collected on uncoated glass slides and stained with methylene blue/azure II (Richardson et al., 1960). Sections were visualized using a Zeisss Axio Imager (Carl Zeiss AG, Jena, Germany).

### 4.10 Scanning electron microscopy

For scanning electron microscopy 3 week old βB1-CTGF6 and wildtype mice were anesthetized with CO_2_ and euthanized by atlanto-occipital dislocation on P21. Afterwards eyes were enucleated and fixed in 2.5% glutaraldehyde in 0.1 M cacodylate buffer (pH 7.4) for 24 h. Next, eyes were washed with four times with 0.1 M cacodylate buffer. Afterwards eyes were treated with 1% OsO4 in 0.1 M cacodylate buffer for 2 h and additionally washed three times with 0.1 M cacodylate buffer (pH 7.4). Next, the samples were dehydrated by an ascending ethanol series (50, 70, 80, 90% and 100%), followed by the incubation with an ethanol and acetone (1:1) mixture for 60 min and acetone for 60 min. Afterwards the dehydrated samples were transferred into acetone and critical point dried using CO_2_. The dried samples were mounted on aluminum stubs, gold-coated and examined in a scanning electron microscope (Carl Zeiss DSM 940A, Jena, Germany).

### 4.11 Image analysis

The images were analyzed using ImageJ’s built-in measuring feature (Wayne Rasband, formerly National Institutes of Health, Bethesda, MD, United States). The area of the entire anterior chamber angle (ciliary body, outflow region and sclera) of the section and separately the outflow area, consisting of the trabecular meshwork and Schlemm`s canal endothelium, was calculated and the amount of area emitting fluorescent signal for pSmad1/5/8 and BMP4 within the outlines was determined by a standardized macro routine consisting of ImageJ’s color threshold plugin and particle analyzer. The resulting values were used to calculate the percentage of area within the outlines. These measurements were performed for each individual specimen after calibrating ImageJ with the scale bar. The resulting data was analyzed using GraphPad Prism 9.5.1 (GraphPad Software, Boston, MA, United States).

### 4.12 Statistical analysis

Western blot, real-time RT-PCR and immunohistochemical analyses was repeated at least three independent times with RNA and proteins from individual HTM-N cells samples and individual anterior eye segment mouse tissue. Each real-time RT-PCR analysis was performed in triplicates. Repeated measurements were neither performed for Western blot nor real-time RT-PCR analyses. The data is represented as mean ± SEM or otherwise stated in the figure legends. Statistical analysis was performed with GraphPad Prism 9.5.1. The statistical test used for each experiment is indicated in the figure legend of the respective experiment. For comparison of two groups the Mann-Whitney test was used. For comparison of two or more groups the Kruskal–Wallis or one-way ANOVA tests was used.

## Data Availability

The raw data supporting the conclusion of this article will be made available by the authors, without undue reservation.
