# Supplementary material for: CCN2/CTGF tip the balance of growth factors towards TGF-β2 in primary open-angle glaucoma
Source: Front Mol Biosci. 2023 May 11;10:1045411. doi: 10.3389/fmolb.2023.1045411 (PMC10210157; doi:10.3389/fmolb.2023.1045411)
Supplement: Supplementary file 1 [file DataSheet1.PDF]

Supplementary Material

1 Supplementary Methods

The levels of CCN2/CTGF in lenses of our transgenic βB1-CTGF mouse lines were assessed by northern blot (Supplementary figure 1). Total RNA was isolated at postnatal day 11 as described. Northern blot analysis were performed as described previously (Junglas, Kuespert et al. 2012).

Vasculature of the ciliary body of wildtype and βB1-CTGF1 mice was analyzed by immunohistochemical staining against CD31 on whole mounts of the ciliary body (Supplementary figure 2).

2 Supplementary Figures

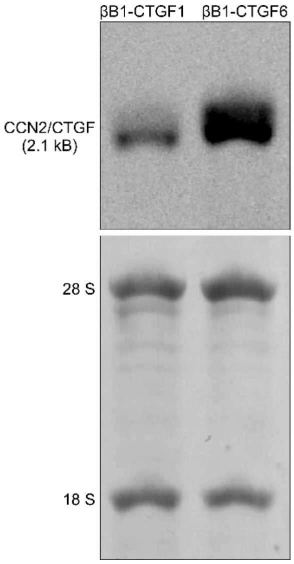

Supplementary Figure 1. Analysis of CCN2/CTGF overexpression in transgenic βB1-CTGF mouse lines. Northern blot analysis for CCN2/CTGF mRNA in the lens of βB1-CTGF1 and βB1-

CTGF6 mice showed a 3.8 fold higher CCN2/CTGF expression in the βB1-CTGF6 mouse line, compared to βB1-CTGF1 mice. Methylene blue staining used to confirm equal loading.

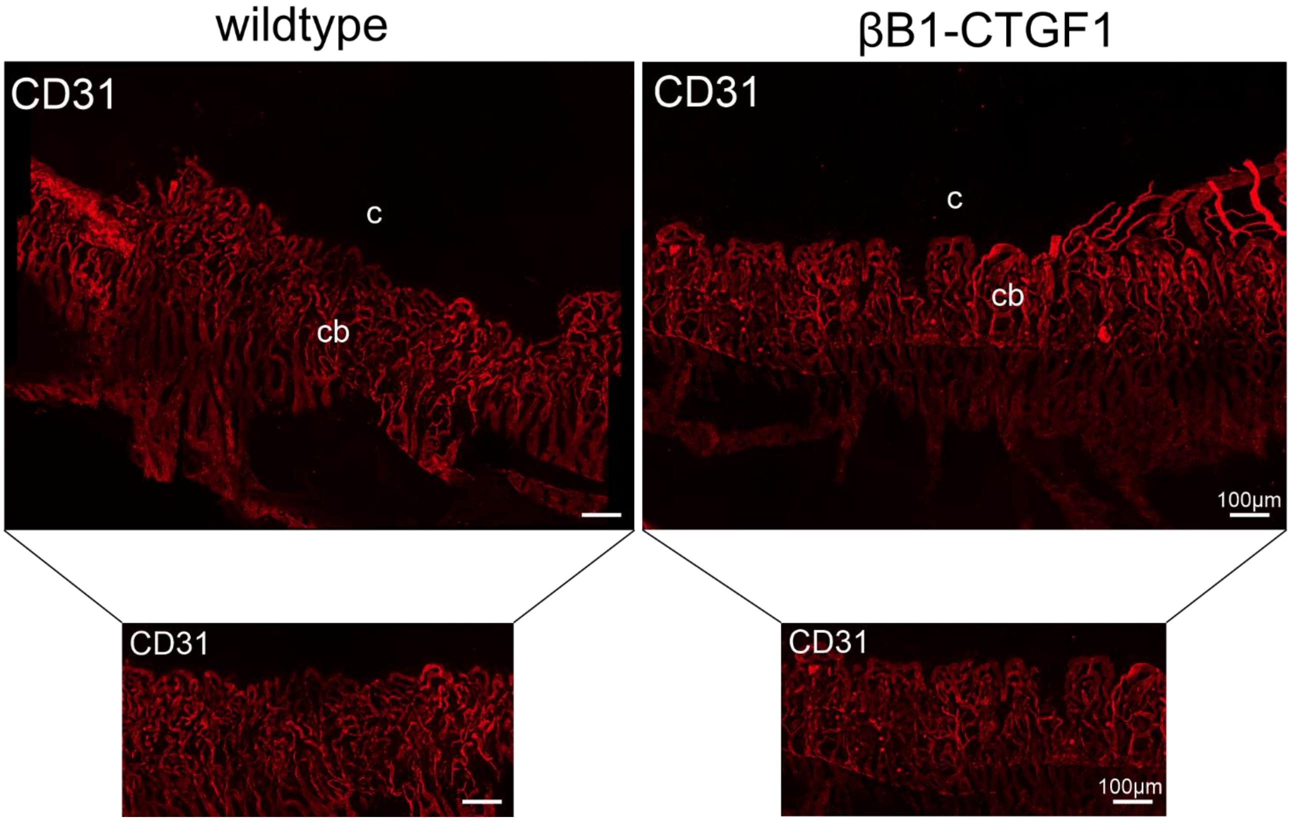

Supplementary Figure 2. Immunohistochemical staining of the ciliary body vasculature in 4 week old wildtype and βB1-CTGF1 mice. Ciliary body vasculature is stained by CD31 (red) on whole

mounts of the ciliary body. βB1-CTGF1 mice show no differences in the formation of the vascular plexus in the ciliary body processes compared to wildtype littermates. c: Cornea, cb: ciliary body; n=3.

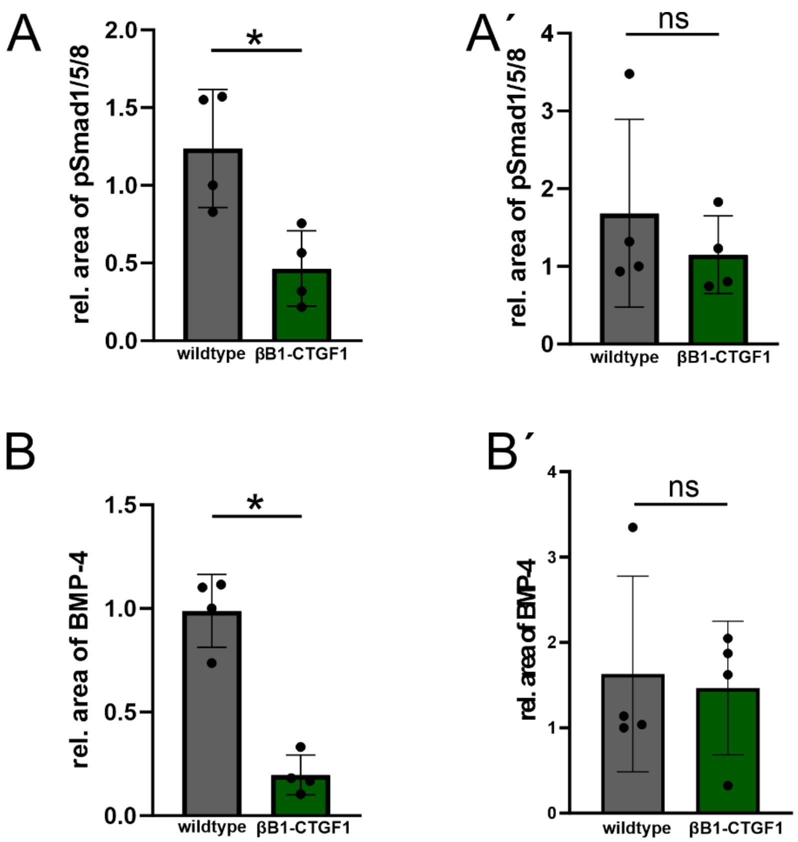

Supplementary Figure 3. Quantification of pSmad1/5/8 and BMP-4 positive area in 2 month old βB1-CTGF1 mice and wildtype mice. (A) Quantification of pSmad1/5/8 positive area in 2 month old βB1-CTGF1 mice and wildtype mice in the outflow area, including the trabecular meshwork and Schlemm's canal endothelium. pSmad1/5/8 positive area was significantly reduced in βB1-CTGF1 mice compared to wildtype littermates. **(A')** Quantification of pSmad1/5/8 positive area in 2 month old βB1-CTGF1 mice and wildtype mice in the entire chamber angle showed no difference in βB1-CTGF1 mice compared to wildtype littermates. **(B)** Quantification of BMP-4 positive area in 2 month old βB1-CTGF1 mice and wildtype mice in the outflow area, including the trabecular meshwork and Schlemm's canal endothelium. BMP-4 positive area was significantly reduced in βB1-CTGF1 mice compared to wildtype littermates. **(B')** Quantification of BMP-4 positive area in 2 month old βB1-CTGF1 mice and wildtype mice in the entire chamber angle showed no difference in βB1-CTGF1 mice compared to wildtype littermates. ns: not significant

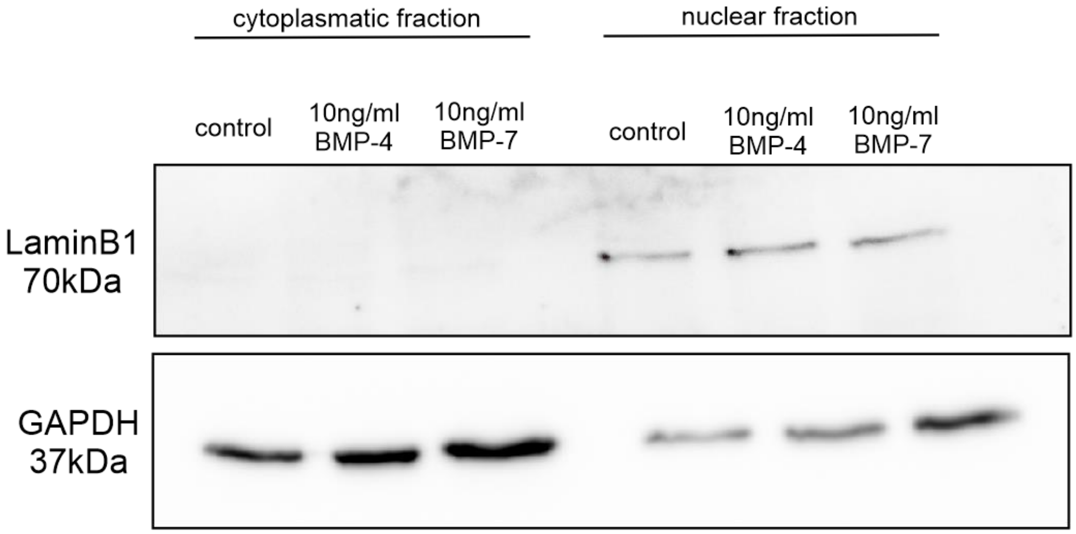

Supplementary Figure 4. Verification of purity of cellular fractions isolated from BMP-4 and BMP-7 treated HTM-N cells. Western blot was performed including the cytoplasmatic and nuclear fraction for LaminB1 (loading control nuclear fraction) and GAPDH (loading control cytoplasmatic fraction). Signal for LaminB1 was only detected in the nuclear fraction and GAPDH could be detected in both fractions.

Junglas, B., S. Kuespert, A. A. Seleem, T. Struller, S. Ullmann, M. Bosl, A. Bosserhoff, J. Kostler, R. Wagner, E. R. Tamm and R. Fuchshofer (2012). "Connective tissue growth factor causes glaucoma by modifying the actin cytoskeleton of the trabecular meshwork." Am J Pathol **180**(6): 2386-2403.
